# Predicting Benefit of Neoadjuvant Chemotherapy and Elective Nodal Irradiation in Pancreatic Adenocarcinoma: A Supervised Machine Learning Approach

**DOI:** 10.1002/cam4.71447

**Published:** 2025-12-05

**Authors:** Garrett K. Harada, Jino Park, Nicholas Peterson, Akul Munjal, David Imagawa, Zeljka Jutric, Farshid Dayyani, Jennifer Valerin, David S. Hong, Steven N. Seyedin

**Affiliations:** ^1^ Department of Radiation Oncology Chao Family Comprehensive Cancer Center University of California Irvine California USA; ^2^ Department of Surgical Oncology Chao Family Comprehensive Cancer Center University of California Irvine California USA; ^3^ Department of Medical Oncology Chao Family Comprehensive Cancer Center University of California Irvine California USA; ^4^ Department of Radiation Oncology Helen Diller Family Comprehensive Cancer Center University of California San Francisco San Francisco California USA

**Keywords:** chemotherapy, elective nodal irradiation, machine learning, neoadjuvant, pancreatic ductal adenocarcinoma

## Abstract

**Background:**

The relative benefit of neoadjuvant therapies remains controversial for patients with (borderline) resectable pancreatic ductal adenocarcinoma (PDAC). The purpose of this study was to create a model to predict response to multiagent neoadjuvant chemotherapy (NAC) followed by radiotherapy with elective nodal irradiation (ENI).

**Methods:**

Using the National Cancer Database (NCDB), we identified patients with cT1‐4N0M0 PDAC diagnosed between 2006 and 2020 treated with multiagent NAC, radiation with ENI, followed by curative resection with nodal dissection. A LASSO logistic regression model was used to predict ypN0 status, with generation of a nomogram and assessment of outcomes in training and testing cohorts. Secondary endpoints of negative‐margin resection and overall survival were also examined. Out‐of‐sample predictions were then made on a separate ENI‐naïve cohort, with similar assessments of selected outcomes. The threshold for statistical significance was set to *p* < 0.05.

**Results:**

A total of 1053 patients were identified with a median age of 64.0 years (IQR = 57–70 years). The final model included pancreatic body tumor location, clinical T stage, time from diagnosis to radiation therapy and surgery, ENI dose, and duration of NAC, among others. Patients predicted for treatment response were more likely to be ypN0 (71.5% vs. 29.7%, *p* < 0.001), had more R0 resections (87.3% vs. 62.6%, *p* < 0.001), and improved OS after accounting for competing risks of perioperative death (SHR = 0.64, 95% CI = 0.46–0.89, *p* = 0.008). A similar significant trend was noted in the ENI‐naïve cohort (*N* = 1258). Model AUC was 0.718 and 0.725 in training and testing cohorts, respectively.

**Conclusions:**

Using a machine learning approach, we define a nomogram capable of predicting treatment response to multiagent NAC followed by radiotherapy with or without ENI. Patients selected by this model had higher rates of ypN0, higher R0 resection rates, and improved OS.

## 1 Introduction

Despite advances in oncologic therapy, complete surgical resection remains the most durable curative option for non‐metastatic pancreatic ductal adenocarcinoma (PDAC). Unresected PDAC has a particularly poor prognosis with median overall survival estimates less than 24 months [1, 2, 3]. As such, modern treatment paradigms have focused on promoting a margin‐negative (R0) resection, with neoadjuvant therapy traditionally reserved for those with borderline resectable disease [4]. Many recent or ongoing efforts have focused on variations utilizing multiagent neoadjuvant chemotherapy (NAC) followed by consideration of (chemo)radiotherapy with or without elective nodal irradiation (ENI) [1, 3, 5]. However, there exists significant heterogeneity in findings from modern neoadjuvant trials, with ongoing debate over the regimen and length of induction chemotherapy, as well as the utility of neoadjuvant radiotherapy with or without ENI.

As a result, for borderline resectable PDAC, national guidelines do not present a strong recommendation for a particular neoadjuvant regimen off‐trial [4]. This highlights one of the many challenges in the management of PDAC, as it remains unclear how different neoadjuvant therapies interact and impact clinical outcomes. The purpose of this study was to derive a nomogram to quantify the effects of neoadjuvant approaches consisting of multiagent NAC and radiotherapy with or without ENI in selecting patients who have favorable responses to therapy.

## 2 Materials and Methods

### 2.1 Patient Cohort Selection

**FIGURE 1:**
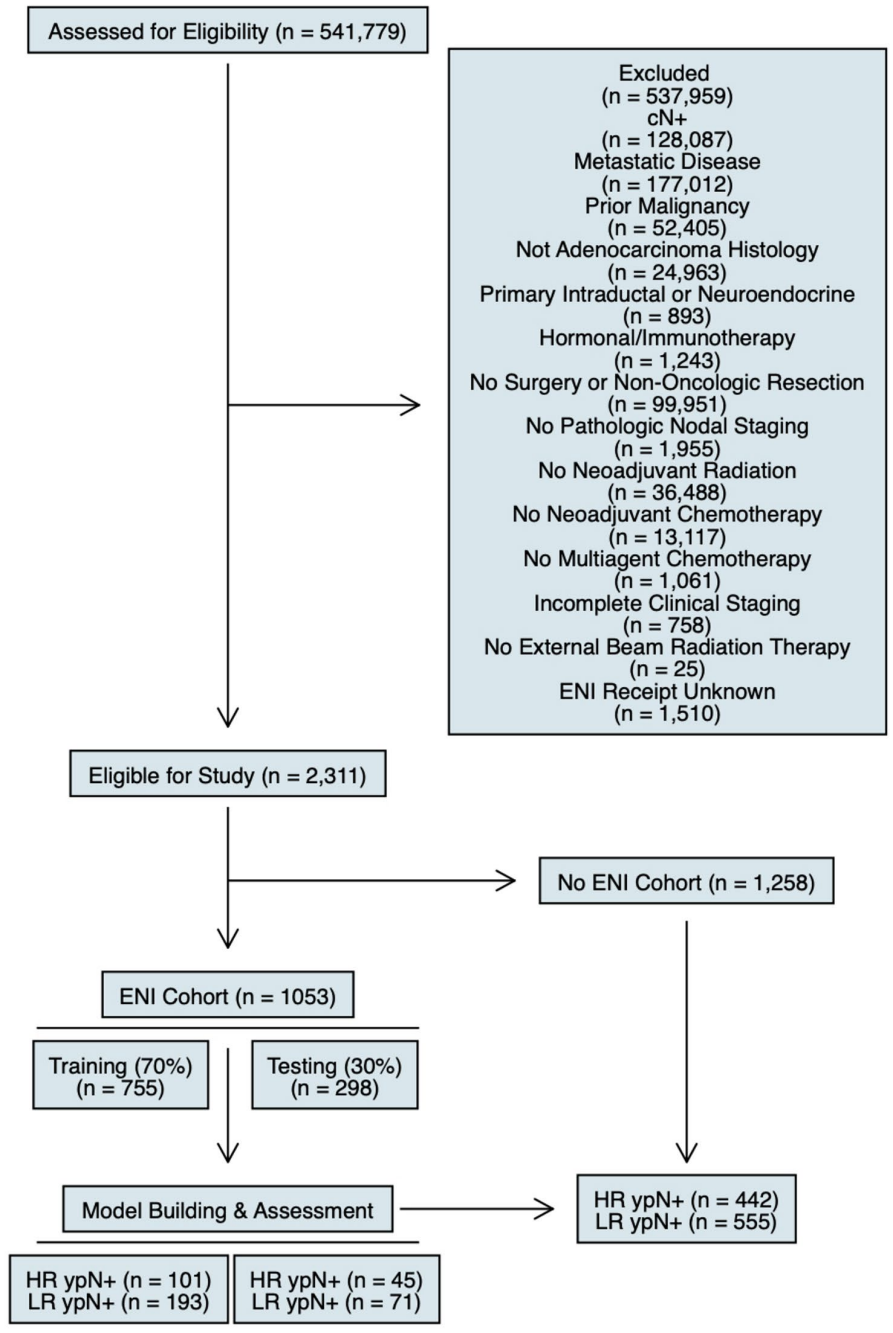
Patient selection flowchart. Flowchart detailing inclusion and exclusion criteria to define ENI and ENI‐naïve cohorts. The final model for ypN status was defined in those receiving neoadjuvant chemotherapy and radiotherapy with ENI followed by out‐of‐sample application to the ENI‐naïve cohort to identify both patients at high and low risk of ypN+ disease. ENI, elective nodal irradiation; HR, high risk; LR, low risk.

Using the National Cancer Database (NCDB), we identified all patients diagnosed with cT1‐4N0M0 pancreatic adenocarcinoma between 2006 and 2020. Patients selected received multiagent NAC, followed by external beam radiation therapy with ENI and definitive surgery with nodal dissection. All patients deemed to have receipt of ENI were required to receive a standardized ENI dose ranging from a biologically effective dose (α/ß = 10 Gy; BED10) of 37.5 Gy (25 Gy in 5 fractions) up to 53.1 Gy (45 Gy in 25 fractions) [5, 6]. Patients were otherwise excluded for a history of prior malignancy, incomplete clinical or pathologic staging, or for primary intraductal or neuroendocrine tumors (Figure 1).

Baseline characteristics were collected for each patient with inclusion of age, sex, race, insurance status, treatment facility type (Community, Comprehensive Community, Academic, Integrated Cancer Network Program), and Charlson‐Deyo score. The Charlson‐Deyo score is a validated metric that measures mortality risk from a select number of medical comorbidities [7, 8]. Tumor features were also recorded and included tumor location, histologic grade, clinical and pathologic TNM stage (AJCC 6th through 8th editions), tumor size (cm), number of lymph nodes examined and metastatically involved, lymphovascular invasion, and baseline CA 19‐9 (U/mL). Treatment variables were collected and included time from diagnosis to NAC, radiation, and/or surgery (days), length of NAC delivery (weeks), type of oncologic resection, radiation dose and fractions delivered to the primary tumor and regional lymph nodes, and radiation technique (3‐D conformal [3DCRT] versus intensity modulated radiation therapy [IMRT]). All radiation doses were recorded using a BED10.

### 2.2 Endpoints

Given the objective of quantifying the effects of NAC and radiotherapy with ENI, complete pathologic nodal response (ypN0) from this treatment regimen was selected as the primary outcome of interest. Other secondary outcomes were also examined including overall survival (OS), surgical margin status (positive versus negative) and response of primary tumor, defined by the change in clinical to pathologic T‐stage, where those with stable to decreased primary tumor involvement were classified as responsive to treatment.

### 2.3 Statistical Analysis

All statistical analysis was performed using Stata v18.0 (StataCorp, College Station, TX). Collected variables were summarized using descriptive statistics and assessed for differences between those with ypN0 and post‐treatment pathologic nodal disease (ypN+). Categorical variables were compared using chi‐square with continuous variables assessed using two‐sided t‐tests or Wilcoxon rank sum tests depending on normality. All variables were then assessed for utility in baseline prediction of ypN0, such that selected features could be defined prior to surgical resection. This included AJCC staging edition given considerations of differences in TNM staging criterion across the study interval. Variables were then assessed for missingness of data, where those with a missing data fraction of 40% or higher were removed from model consideration. Prevalence and patterns of missing data information are summarized in (File S1). Further, interaction terms were generated between selected variables to assess potential effect modification between treatment parameters and included weeks of NAC with ENI BED10, clinical T‐stage, duration of radiation therapy treatment, as well as ENI BED10 with clinical T‐stage.

Following selection and a 70:30 training to testing data split, variables and candidate interaction terms were entered into a LASSO logistic regression model with control of possible clustering by treating institution. Model hyperparameters were selected using 10 folds of cross‐validation with subsequent derivation of coefficients for selected covariates. The model was then used to predict the probability of ypN0 in both testing and training cohorts. A probability threshold was established using Youden's index, where those above and below 52.8% probability were deemed low‐ and high‐risk to have ypN+ disease after NAC and radiotherapy with ENI, respectively. Model discrimination was assessed using the area under the receiver operating characteristic curves (AUC) with goodness‐of‐fit measured with calibration plots.

Secondary outcomes of OS, surgical margin status, and primary tumor response to therapy were similarly assessed against ypN risk group using chi‐square. OS was summarized using the Kaplan–Meier method and log‐rank test, with the surveillance interval defined from the date of definitive surgery and censored to the date of final follow‐up or death. Additional Fine‐Gray models and cumulative incidence functions were also used to assess non‐operative mortality, controlling for competing risks of postoperative death within 90 days.

Lastly, a separate ENI‐naïve cohort meeting all other inclusion criteria was selected for purposes of assessing the applicability of the model for those receiving multiagent NAC and radiotherapy without ENI (Figure 1). Youden's index was again performed to select a new threshold given the omission of ENI, with those above and below a probability of 33.1% selected to be low‐ and high‐risk of ypN+ disease, respectively. All secondary outcomes of OS, primary tumor downstaging, and surgical margin status were similarly assessed by model risk group stratification. The threshold for statistical significance was set to *p* < 0.05.

## 3 Results

### 3.1 Patient and Tumor Characteristics

A total of 1053 patients were identified for study with a median age of 64 years (IQR = 57–70 years). Tables 1 and 2 display differences in baseline patient and treatment characteristics. Patients with ypN+ disease were more likely to be male (*p* = 0.029), treated at comprehensive community cancer programs (*p* = 0.040), and more frequently diagnosed beginning in 2016 (*p* < 0.001). In contrast, those who were ypN0 had a greater incidence of cT4 disease when compared to those who were ypN+ (*p* = 0.002). Patients with ypN+ disease started treatment later than those with ypN0 disease, with later initiation of NAC (*p* = 0.010) and radiation therapy (*p* < 0.001) after diagnosis. Further, patients with ypN0 disease were most likely to receive 21 or more weeks of NAC (*N* = 429/600, 71.9%), while those with ypN+ more often received 16 weeks or fewer (*N* = 74/453, 16.4%; *p* < 0.001). Those with ypN+ demonstrated a greater incidence of positive margins (ypN+ = 30.9%, ypN0 = 12.8%; *p* < 0.001) and pT3 disease (ypN+ = 76.9%, ypN0 = 49.4%, *p* < 0.001).

**TABLE 1:** Baseline patient characteristics by ypN status.

	ypN0	ypN+	Total	*p* value
*N*	600 (57.0%)	453 (43.0%)	1053 (100.0%)	
Age (years) [IQR]	63.000 [57.0–69.0]	64.000 [57.0–70.0]	64.000 [57.0–70.0]	0.395
Sex				
Male	281 (46.8%)	243 (53.6%)	524 (49.8%)	**0.029**
Female	319 (53.2%)	210 (46.4%)	529 (50.2%)	
Race				
American Indian	1 (0.2%)	3 (0.7%)	4 (0.4%)	0.802
Asian Indian	2 (0.3%)	1 (0.2%)	3 (0.3%)	
Asian Indian or Pakistani, NOS	1 (0.2%)	1 (0.2%)	2 (0.2%)	
Black	57 (9.6%)	42 (9.4%)	99 (9.5%)	
Chinese	3 (0.5%)	0 (0.0%)	3 (0.3%)	
Japanese	0 (0.0%)	1 (0.2%)	1 (0.1%)	
Korean	3 (0.5%)	1 (0.2%)	4 (0.4%)	
Other	3 (0.5%)	3 (0.7%)	6 (0.6%)	
Other Asian, NOS	6 (1.0%)	4 (0.9%)	10 (1.0%)	
Vietnamese	1 (0.2%)	1 (0.2%)	2 (0.2%)	
White	515 (87.0%)	392 (87.3%)	907 (87.1%)	
Insurance				
Uninsured	43 (7.3%)	24 (5.4%)	67 (6.5%)	0.797
Private insurance/managed care	262 (44.4%)	206 (46.1%)	468 (45.1%)	
Medicaid	6 (1.0%)	5 (1.1%)	11 (1.1%)	
Medicare	10 (1.7%)	7 (1.6%)	17 (1.6%)	
Other government	269 (45.6%)	205 (45.9%)	474 (45.7%)	
Year of diagnosis				
2006	14 (2.3%)	5 (1.1%)	19 (1.8%)	**< 0.001**
2007	11 (1.8%)	6 (1.3%)	17 (1.6%)	
2008	20 (3.3%)	7 (1.5%)	27 (2.6%)	
2009	22 (3.7%)	9 (2.0%)	31 (2.9%)	
2010	20 (3.3%)	11 (2.4%)	31 (2.9%)	
2011	31 (5.2%)	20 (4.4%)	51 (4.8%)	
2012	39 (6.5%)	22 (4.9%)	61 (5.8%)	
2013	41 (6.8%)	25 (5.5%)	66 (6.3%)	
2014	60 (10.0%)	38 (8.4%)	98 (9.3%)	
2015	85 (14.2%)	67 (14.8%)	152 (14.4%)	
2016	107 (17.8%)	64 (14.1%)	171 (16.2%)	
2017	94 (15.7%)	87 (19.2%)	181 (17.2%)	
2018	28 (4.7%)	32 (7.1%)	60 (5.7%)	
2019	15 (2.5%)	35 (7.7%)	50 (4.7%)	
2020	13 (2.2%)	25 (5.5%)	38 (3.6%)	
Treatment facility type				
Community cancer program	15 (2.5%)	11 (2.4%)	26 (2.5%)	**0.040**
Comprehensive community cancer program	100 (16.9%)	106 (23.6%)	206 (19.8%)	
Academic/research program	352 (59.4%)	234 (52.1%)	586 (56.2%)	
Integrated network cancer program	126 (21.2%)	98 (21.8%)	224 (21.5%)	
Baseline CA 19‐9 (U/mL) [IQR]	980.000 [242.0–980.0]	980.000 [417.0–980.0]	980.000 [302.0–980.0]	0.425
Tumor primary location				
Head	447 (74.5%)	362 (79.9%)	809 (76.8%)	0.185
Body	65 (10.8%)	36 (7.9%)	101 (9.6%)	
Tail	23 (3.8%)	14 (3.1%)	37 (3.5%)	
Other	17 (2.8%)	6 (1.3%)	23 (2.2%)	
Overlapping	33 (5.5%)	20 (4.4%)	53 (5.0%)	
Unspecified	15 (2.5%)	15 (3.3%)	30 (2.8%)	
Grade				
1	41 (15.1%)	20 (9.2%)	61 (12.4%)	0.147
2	150 (55.1%)	118 (54.1%)	268 (54.7%)	
3	78 (28.7%)	78 (35.8%)	156 (31.8%)	
4	3 (1.1%)	2 (0.9%)	5 (1.0%)	
AJCC TNM edition				
6th Edition	67 (11.2%)	27 (6.0%)	94 (8.9%)	**< 0.001**
7th Edition	477 (79.5%)	334 (73.7%)	811 (77.0%)	
8th Edition	56 (9.3%)	92 (20.3%)	148 (14.1%)	
Clinical T stage				
cT1	33 (5.5%)	24 (5.3%)	57 (5.4%)	**0.002**
cT2	150 (25.0%)	159 (35.2%)	309 (29.4%)	
cT3	278 (46.3%)	193 (42.7%)	471 (44.8%)	
cT4	139 (23.2%)	76 (16.8%)	215 (20.4%)	

*Note:* Baseline demographic and tumor characteristics are displayed by ypN status. Comparisons between cohorts are performed using a combination of chi‐square and Wilcoxon rank sum for categorical and continuous variables, respectively. Bolded values indicate statistical significance at *p* < 0.05.

Abbreviations: IQR, interquartile range; NOS, not otherwise specified.

**TABLE 2:** Treatment and Pathology Characteristics by ypN Status.

	ypN0	ypN+	Total	*p* value
*N*	600 (57.0%)	453 (43.0%)	1053 (100.0%)	
Time from diagnosis to systemic therapy (days) [IQR]	26.0 [18.0–36.0]	28.0 [19.0–40.0]	27.0 [19.0–37.0]	**0.010**
Weeks of NAC delivered				
≤ 4	< 5 (0.8%)	11 (2.4%)	16 (1.5%)	**< 0.001**
5–8	1 (0.2%)	4 (0.9%)	5 (0.5%)	
9–12	30 (5.0%)	57 (12.6%)	87 (8.3%)	
13–16	53 (8.9%)	74 (16.4%)	127 (12.1%)	
17–20	79 (13.2%)	72 (16.0%)	151 (14.4%)	
≥ 21	429 (71.9%)	233 (51.7%)	662 (63.2%)	
Time from diagnosis to radiation therapy (days) [IQR]	136.0 [104.0–182.0]	165.5 [118.0–233.0]	146.0 [110.0–200.0]	**< 0.001**
Radiation technique				
3DCRT	58 (9.7%)	47 (10.4%)	105 (10.0%)	0.704
IMRT	200 (33.3%)	169 (37.3%)	369 (35.0%)	0.181
Duration of radiation therapy (days) [IQR]	38.0 [24.0–41.0]	37.5 [25.0–40.0]	38.0 [24.0–41.0]	0.467
BED10 delivered to primary tumor (Gy) [IQR]	53.1 [48.0–59.5]	53.1 [48.0–59.5]	53.1 [48.0–59.5]	0.560
BED10 delivered to elective lymph nodes (Gy) [IQR]	53.1 [46.8–53.1]	53.1 [48.0–53.1]	53.1 [48.0–53.1]	0.421
Time from diagnosis to definitive surgery (days) [IQR]	202.0 [157.0–244.0]	175.0 [136.0–223.0]	189.0 [147.0–233.0]	**< 0.001**
Pathologic tumor size (mm) [IQR]	31.0 [25.0–40.0]	32.0 [25.0–40.0]	32.0 [25.0–40.0]	0.247
Number of lymph nodes metastatically involved [IQR]	0.0 [0.0–0.0]	2.0 [1.0–4.0]	0.0 [0.0–2.0]	**< 0.001**
Number of lymph nodes dissected [IQR]	15.0 [10.0–21.0]	18.0 [13.0–26.0]	16.0 [11.0–23.0]	**< 0.001**
Surgery type				
Partial pancreatectomy	80 (13.3%)	40 (8.8%)	120 (11.4%)	0.070
Partial pancreatectomy and duodenectomy	39 (6.5%)	31 (6.8%)	70 (6.6%)	
Partial pancreatectomy and duodenectomy without distal/partial gastrectomy	52 (8.7%)	46 (10.2%)	98 (9.3%)	
Partial pancreatectomy and duodenectomy with distal/partial gastrectomy (whipple)	327 (54.5%)	236 (52.1%)	563 (53.5%)	
Total pancreatectomy	20 (3.3%)	18 (4.0%)	38 (3.6%)	
Total pancreatectomy and subtotal gastrectomy	44 (7.3%)	54 (11.9%)	98 (9.3%)	
Extended pancreatoduodenectomy	38 (6.3%)	28 (6.2%)	66 (6.3%)	
Margin status				
Positive	76 (12.8%)	135 (30.9%)	211 (20.5%)	**< 0.001**
Negative	518 (87.2%)	302 (69.1%)	820 (79.5%)	
Pathologic T stage				
pT0	33 (6.4%)	0 (0.0%)	33 (3.8%)	**< 0.001**
pT1	121 (23.6%)	21 (5.8%)	142 (16.2%)	
pT1a	2 (0.4%)	0 (0.0%)	2 (0.2%)	
pT1b	1 (0.2%)	1 (0.3%)	2 (0.2%)	
pT1c	2 (0.4%)	1 (0.3%)	3 (0.3%)	
pT2	82 (16.0%)	52 (14.3%)	134 (15.3%)	
pT3	253 (49.4%)	279 (76.9%)	532 (60.8%)	
pT4	17 (3.3%)	9 (2.5%)	26 (3.0%)	
pTis	1 (0.2%)	0 (0.0%)	1 (0.1%)	
Pathologic N stage				
pN0	600 (100.0%)	0 (0.0%)	600 (57.0%)	**< 0.001**
pN1	0 (0.0%)	431 (95.1%)	431 (40.9%)	
pN2	0 (0.0%)	22 (4.9%)	22 (2.1%)	
Pathologic M stage				
pM0	263 (100.0%)	188 (99.5%)	451 (99.8%)	0.238
pM1	0 (0.0%)	1 (0.5%)	1 (0.2%)	
Lymphovascular invasion	75 (19.6%)	171 (54.8%)	246 (35.4%)	**< 0.001**
Primary tumor treatment response	214 (82.0%)	141 (75.0%)	355 (79.1%)	0.072

*Note:* Details for administered neoadjuvant chemotherapy, radiotherapy, and surgery with accompanying pathologic findings are displayed by ypN status. Comparisons between cohorts are performed using a combination of chi‐square and Wilcoxon rank sum for categorical and continuous variables, respectively. Bolded values indicated statistical significance at *p* < 0.05.

Abbreviations: 3DCRT, 3‐D conformal radiation therapy; BED10, Biologically effective dose using an α/β = 10 Gy; IMRT, Intensity modulated radiation therapy; IQR, Interquartile range; NAC, Neoadjuvant chemotherapy; NOS, Not otherwise specified.

### 3.2 Final Model for Prediction of ypN0

**FIGURE 2:**
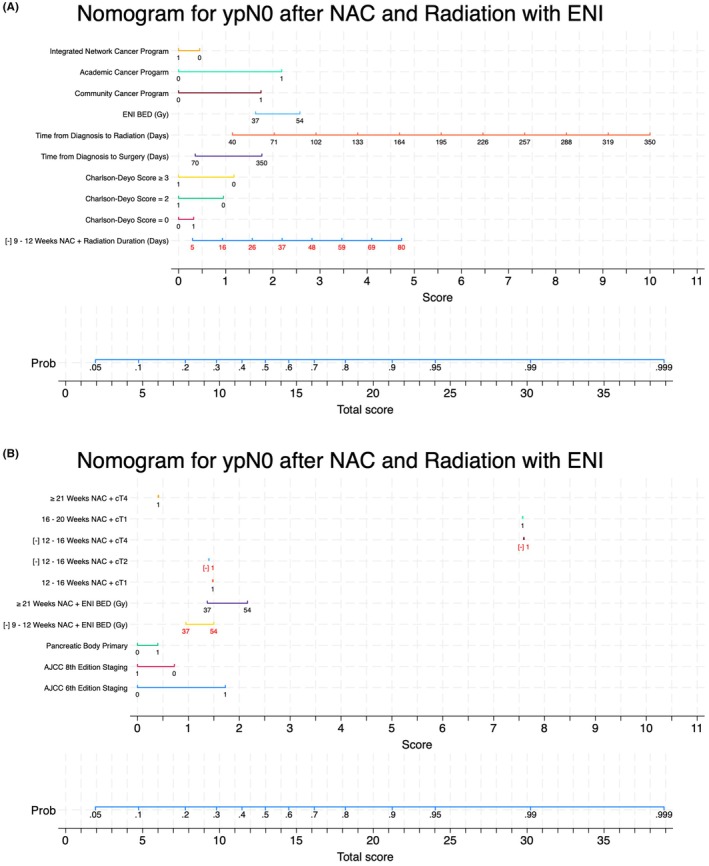
(A, B) Nomogram for ypN Status after neoadjuvant chemotherapy and radiotherapy with ENI. Selected features for predicting risk of ypN0 after neoadjuvant chemotherapy and radiotherapy with ENI are displayed with associated ranges and point values. Nomogram was generated using simulated data with exclusion of female sex given a near zero effect size estimate. To account for this, all scores should have an additive correction factor equal to 0.08 prior to selecting actual probability of ypN0. Scores denoted by red text and a “[−]” should be subtracted while others are additive. Patients with scores > 13.5 correspond to the threshold probability of 52.8% defined in this study, where those above and below were low‐ and high‐risk for ypN+, respectively.

The final model selected included female sex, Charlson‐Deyo score, time from diagnosis to radiation therapy and surgery, treatment facility type (Community, Academic, Integrated Cancer Network Program), pancreatic body tumor location, AJCC 6th and 8th edition staging criterion, and multiple interaction terms between the duration of chemotherapy, radiation treatment parameters, and clinical T stage (Table 3). The final model suggests those with greater comorbidity, delays in radiation therapy initiation, treatment at an integrated network cancer program, and AJCC 8th edition staging were more likely to be ypN+ after neoadjuvant therapy. Further, model interaction terms suggest a dependence between the weeks of NAC and underlying clinical T‐stage, ENI BED10, and radiation therapy duration. Specifically, those with cT4 disease were more prone to ypN+ disease unless given 21 or more weeks of NAC, while those with cT1 disease only required 12–16 weeks to achieve ypN0. In addition, the number of cycles of NAC seemed to have a synergistic effect with ENI, as those receiving 21 or more weeks were likely to be ypN0 with higher ENI doses. While female sex was associated with ypN0 after neoadjuvant therapy, the magnitude of the effect was minimal and was subsequently dropped from the nomogram for simplicity (Figure 2A,B). This led to the adjustment of the nomogram by the introduction of a correction factor of approximately 0.08 points.

**TABLE 3:** Final LASSO logistic regression model for ypN status after neoadjuvant chemotherapy and radiotherapy with ENI.

Covariate	β coefficient	Minimum point value^b^	Maximum point value^b^
Demographics			
Female Sex^a^	0.004	—	—
Charlson‐Deyo Score			
≥ 3	−0.261	0 (Score ≥ 3)	1.2 (Score < 3)
2	−0.292	0 (Score = 2)	1 (Score ≠ 2)
0	0.104	0 (Score ≠ 0)	0.3 (Score = 0)
Treating facility			
Integrated network cancer program	−0.113	0 (Treated)	0.4 (Treated Elsewhere)
Academic cancer program	0.573	0 (Treated Elsewhere)	2.2 (Treated)
Community cancer program	0.538	0 (Treated Elsewhere)	1.7 (Treated)
Tumor features			
Pancratic body primary location	0.097	0 (Head, Tail)	0.4 (Body)
AJCC TNM staging			
6th edition	0.478	0 (7th or 8th Edition)	1.7 (6th Edition)
8th edition	−0.198	0 (8th Edition)	0.7 (6th or 7th Edition)
Treatment features			
ENI BED10 (Gy)	0.011	1.6 (37.5 Gy)	2.5 (54 Gy)
Time from diagnosis to radiation (Days)	−0.007	1.1 (40 days)	10 (350 days)
Time from diagnosis to surgery (days)	0.001	0.4 (70 days)	1.8 (350 days)
Treatment interaction terms			
Number of cycles of NAC + radiation duration (days)			
3 cycles	−0.014	−4.7 (80 days)	−0.3 (5 days)
Number of cycles of NAC + ENI BED10 (Gy)			
3 cycles	−0.008	−1.5 (54 Gy)	−1.0 (37.5 Gy)
6 cycles	0.010	1.4 (37.5 Gy)	2.2 (54 Gy)
Number of cycles of NAC + clinical T stage			
4 Cycles + cT1	0.246	0 (cT2‐4)	1.5 (cT1)
4 Cycles + cT2	−0.350	−1.4 (cT2)	0 (cT1, cT3‐4)
4 Cycles + cT4	−1.962	−7.6 (cT4)	0 (cT1‐3)
5 Cycles + cT1	2.138	0 (cT2‐4)	7.6 (cT1)
6 Cycles + cT4	0.102	0 (cT1‐3)	0.4 (cT4)
Correction factor^c^	—	0.08	0.08

*Note:* Final model covariates selected using LASSO logistic regression with 10 folds of cross‐validation. Tuning hyperparameter () was established at 0.024 to derive penalized ß coefficients for covariates with a nonzero effect on ypN status after neoadjuvant chemotherapy and radiotherapy with ENI.

Abbreviations: AJCC TNM staging, Model adjustments made by American Joint Commission on Cancer staging edition employed due to shifts in both cT and cN criterion from the 6th to 8th editions; Charlson‐Deyo Score, Standardized prognostic metric for 10‐year survival using a set of comorbid conditions; ENI BED10, Elective nodal irradiation biologically effective dose using an α/β = 10 Gy. Doses delivered were limited to a BED10 of 37.5 Gy to 54 Gy; NAC, neoadjuvant chemotherapy; time to radiation (days), actual range was 40–350 days; Time to surgery (days), actual range was 70–350 days.

^a^ The effect size of female sex was observed to be orders of magnitude lower than other covariates and was subsequently dropped from the model prior to nomogram derivation.

^b^ Point values represent a scaled measure of the penalized coefficient. Values were calculate using simulated data approximating the distribution of selected covariates within patients randomly assigned to the training cohort. Ranges indicate minimum and maximum values possible given the observed data. Note that where possible, negative coefficients were forced positive with adjustment of all other coefficients in order to facilitate simplicity of calculations.

^c^ Given the decision to drop female sex from the model, a correction factor was introduced for recalibration. The correction factor accounts for a 0.1%–2.0% increase in the probability of ypN0 after neoadjuvant therapy across all cases.

### 3.3 Model Performance for Primary and Secondary Outcomes

Using a threshold probability of 52.8%, patients within the low‐risk group were more likely to be ypN0 (*N* = 138/193, 71.5%) when compared to those deemed high‐risk (*N* = 30/101, 29.7%; *p* < 0.001). A similar distribution was seen within the testing cohort, with 71.8% (*N* = 51/71) of low‐risk patients having ypN0 versus 44.4% (*N* = 20/45) of high‐risk patients (*p* = 0.003). Overall model calibration suggested goodness‐of‐fit with discrimination noting an AUC = 0.718 and AUC = 0.725 in training and testing cohorts, respectively (Table S1, Figure S1).

**FIGURE 3:**
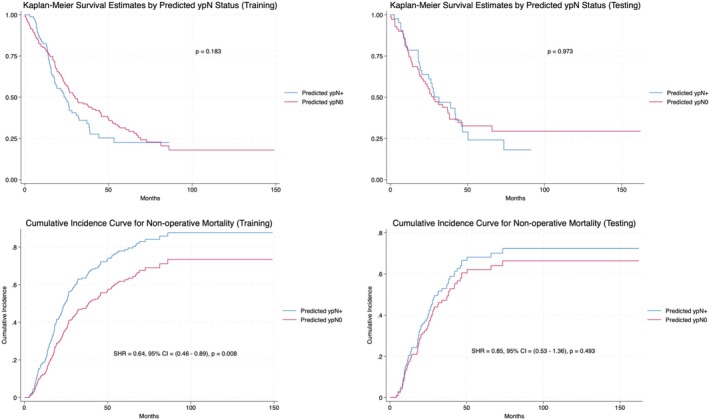
Kaplan–Meier and cumulative incidence curve survival analysis—training and testing cohorts. Kaplan–Meier and cumulative incidence survival curves are displayed for both training (Left) and testing (Right) cohorts. All patients were observed beginning at the date of surgery and censored to the date of final follow‐up or death. *p* values for the log‐rank test are displayed for Kaplan–Meier survival estimates whereas subdistribution hazard ratios, 95% confidence intervals, and Gray's test *p* values are shown for cumulative incidence curves. Competing risks were defined as 90‐day postoperative mortality. The threshold for statistical significance was set to *p* < 0.05. CI, confidence interval; SHR, Subdistribution hazard ratio.

On survival analysis, low‐risk patients had a median OS of 30.2 months (95% CI = 24.3–40.4 months) and 27.8 months (95% CI = 20.2–38.3 months) in training and testing cohorts, respectively. In contrast, high‐risk patients had a median OS of 23.4 months (95% CI = 17.7–30.7 months; *p* = 0.183) and 31.5 months (95% CI = 19.4–45.9 months; *p* = 0.973). Within training data, and after accounting for competing risks of postoperative mortality, low‐risk patients had a significantly lower subdistribution hazard for nonsurgical death when compared to the high‐risk cohort (SHR = 0.64, 95% CI = 0.46–0.89, *p* = 0.008). This was reflected by an improved median OS of 34.8 months (95% CI = 26.5–45.7 months) and 23.4 months (95% CI = 17.7–30.7 months), respectively (Figure 3). A similar improvement in median OS was seen in testing data, though it was nonsignificant between ypN risk groups.

For other secondary outcomes, model stratification for ypN status did not demonstrate any relationship with primary tumor response in both training (*p* = 0.988) and testing cohorts (*p* = 0.212). There was, however, a significant association with surgical margin status, as low‐risk patients had significantly higher rates of negative margin resection at 87.3% and 89.9% for training and testing cohorts, respectively (*p* < 0.001) (Table S1).

### 3.4 Model Application in Multiagent NAC and Radiotherapy Without ENI

A separate ENI‐naïve cohort of 1258 patients was then used for out‐of‐sample predictions to determine if the model could retain utility in the identification of ypN status. Differences between ENI and ENI‐naïve cohorts are as summarized in (Table S2). Patients without ENI were more likely treated beginning in 2018, often received 24 or more weeks of NAC, and had lower clinical and pathologic T staging, likely secondary to changes from the AJCC 7th to 8th editions (*p* < 0.001, for all). The model subsequently identified high‐ and low‐risk for ypN+ cohorts with 43.9% (*N* = 194/442) and 33.7% (*N* = 187/555) cases of ypN+, respectively (*p* = 0.001). Low‐risk patients had a median OS of 37.6 months (95% CI = 30.5—NR) while those deemed high‐risk survived 28.3 months (95% CI = 22.5–33.8 months) (*p* = 0.057). A significant survival benefit was seen for low‐risk patients after accounting for competing risks of postoperative mortality (SHR = 0.73, 95% CI = 0.57–0.93, *p* = 0.012) (Figure S2). Rates of R0 resection were similarly higher for the low‐risk cohort, at 87.2% versus 74.3% for those at high risk of ypN+ (*p* < 0.001), while no significant difference in tumor primary downstaging was noted (*p* = 0.943).

## 4 Discussion

Neoadjuvant therapy in the management of PDAC presents with multiple options reporting conflicting results and modest therapeutic gains. In this manuscript, we attempt to quantify the effects of multiagent NAC and radiation therapy with ENI, insofar as they relate to outcomes of ypN status, R0 resection rate, and OS. Using the NCDB, our model suggests patient comorbidity, clinical T stage, ENI dose delivered, and timeliness of radiation and surgical resection could predict patients likely to demonstrate improved pathologic response from neoadjuvant therapy. Moreover, there appears to be a potential relationship between neoadjuvant treatment modalities, such that those with more advanced disease benefit from more NAC, while similarly, those who receive sufficient chemotherapy doses may derive greater benefit from radiation with ENI. Such findings speak to the heterogeneity in treatment response for PDAC, and could help clarify the role of individual therapies, assist providers in personalizing treatment for individual patients, and also provide further prognostic utility for those eligible for oncologic resection.

To our knowledge, no previous nomograms quantify the effects of neoadjuvant therapies for favorable disease biology in PDAC. Shi et al. utilized the SEER database to create a nomogram predicting 1‐, 2‐, 3‐, and 5‐year OS after surgery for PDAC with consideration of the receipt of chemotherapy and radiation [9]. While their nomogram suggests a uniform survival benefit with both modalities, it does not indicate the sequencing or details of these therapies, such that doses and durations are omitted, and it is unclear if treatment was administered pre‐ and/or post‐operatively. Here we provide further context by specifying how neoadjuvant therapy could be administered to maximize the probability of treatment benefit. Such a tool could be useful in clinical practice, as we provide a means to individualize therapies for patients with PDAC using presenting disease pathology and projected neoadjuvant treatment course. Despite this, we encourage further validation and possible refinement of this model before wide implementation, as there are a number of omitted features that may also be of benefit in predicting treatment response. CA 19‐9 is perhaps the most well‐established metric that is absent due to missing data, and has established clinical use in measuring therapy response. With the available data on hand, we performed a post hoc assessment looking at the interaction between CA 19‐9 and individual nomogram probabilities, finding significantly greater odds of ypN0 with higher CA 19‐9 levels and predicted nomogram risk (OR = 1.02, 95% CI = 1.01–1.03, *p* = 0.003). Speculating, this may suggest the tendency to prescribe longer courses of chemotherapy for elevated measures of CA 19‐9, contributing to pathologic nodal response. Other relationships may be seen with similarly established tumor markers (CA‐125), imaging response to neoadjuvant therapies, or newer commercial assays such as circulating tumor DNA (ctDNA). Pending similar investigations on other clinical metrics, to aid the use of this nomogram we have provided an example clinical scenario described in File S2.

While here we examine a specific neoadjuvant regimen, it is worth noting that other approaches are employed for PDAC, with consideration of omitting radiotherapy due to its controversial benefit. While it is unclear how our findings apply to these other neoadjuvant regimens, here we demonstrate the utility of radiation and ENI, and suggest that its efficacy may be inherently tied to the successful administration of NAC. Specifically, short durations of NAC had more ypN+ disease with longer or dose‐intensified radiotherapy, while longer courses of NAC derived greater benefit from ENI dose escalation. This suggests an interplay between neoadjuvant therapies, and could provide some context for some of the recent controversies surrounding radiation. For example, the Alliance (A021501) phase 2 trial recently reported no benefit with the addition of hypofractionated radiation after 7 cycles of mFOLFIRINOX (14 weeks) when compared to 8 cycles of mFOLFIRINOX alone (16 weeks) [3]. While it is unclear if this difference in NAC duration is critical, our results do suggest longer courses may be beneficial when radiation is to follow. There is otherwise no other data highlighting what may be the appropriate duration of NAC in multimodal treatment settings for PDAC, and emphasizes the need to further investigate how variations in one treatment regimen impact another.

Use of ENI is similarly controversial, as there have been no trials examining its use in PDAC. This has led some organizations to recommend against ENI for PDAC, while proponents raise concerns of marginal and locoregional failures when radiotherapy is reserved for gross disease [4, 5, 10, 11, 12, 13, 14]. Retrospective studies have since documented patterns of locoregional failure for PDAC, where approximately 90% of recurrences may have been addressed with utilization of ENI [14, 15]. Further, there is evidence that an elective dose as low as 25 Gy in 5 fractions may be sufficient to significantly reduce the risk of locoregional progression at 2 years in PDAC [5]. Our nomogram expands upon this, suggesting potential benefit to dose escalation for ENI, as doses approaching a BED10 of 53.1 Gy led to more ypN0 when compared to 37.5 Gy. There is otherwise no prior evidence on dose escalation for ENI in PDAC, and other logistical concerns such as nearby organs at risk may limit significantly higher dose administration. Dose escalation in such settings would likely need to be accomplished via the adoption of newer techniques such as magnetic resonance‐guided radiotherapy and/or with consideration of a longer fractionated course of radiotherapy to allow for lower normal tissue toxicity [5, 6, 16]. Despite this, our model also retained validity when applied to a cohort where ENI is omitted, suggesting further application in clinical practices where radiotherapy is reserved for gross disease. Future work should aim to revalidate these findings in both ENI and ENI‐naïve cohorts to verify such applicability prior to wide implementation in clinical practice.

Our findings are of particular interest as it suggests that changes in the delivery of neoadjuvant treatment could also impact survival in PDAC prior to surgery. Within the training data, patients deemed low‐risk for ypN+ disease had an improvement in their median OS to 34.8 months (vs. 23.4 months) after accounting for competing risks of postoperative mortality. This figure approximates the better median OS estimates for neoadjuvant therapy in PDAC, suggesting that select patients have a favorable prognosis driven by reduced risk of ypN+ disease and increased R0 resection rate [3, 17, 18]. In contrast, this also implies that there is a cohort of patients unable to realize treatment success despite multimodal therapy. Here, our nomogram suggests that these patients have advanced primary disease, are unable to complete an adequate course of NAC, and further risk reduction with radiotherapy and ENI is unfeasible. This distinction highlights the probable heterogeneity in PDAC and identifies that patients who are able to receive adequate neoadjuvant therapy regimens have favorable disease biology. While the precise mechanisms that underlie favorable responses to neoadjuvant treatment are unknown, our nomogram provides a means to identify which patients are likely to have a poor prognosis prior to proceeding to surgery, and may identify a cohort to benefit from clinical trial enrollment for new and investigative therapies but requires prospective validation [19, 20, 21, 22].

This study presents a number of limitations. This includes those common to all retrospective or database studies, such as the potential for selection bias, errors in data entry, problems with missing data (i.e., CA 19‐9), and a lack of individual patient granularity to comprehensively identify potential confounding or effect modification. These effects may be particularly compounded given the LASSO methodology, as such an approach produces a biased estimator to fit a model to the supplied data. While efforts were made to control for heterogeneity in treatment and patient populations as well as clustering due to treating center, there exists the potential that other unmeasured variables could affect relationships between selected covariates and ypN+ disease. Notably, this could include the inability to discern concurrent chemoradiation, delivery of adjuvant or salvage therapies, and lack of resectability assessments, all of which are critical to the modern management of PDAC. This is particularly significant when considering variation in chemotherapy regimens over time, as FOLFIRINOX and gemcitabine/nab‐paclitaxel did not come into practice until after 2010. Despite this, by limiting the cohort to only multiagent regimens, over 90% of the eligible cohort was treated after 2010, and suggests towards selection of patients treated with modern chemotherapy agents. Similar thoughts may also apply to radiation therapy, particularly when considering the use of simultaneous integrated boost (SIB) techniques over time. At present, the convention for the NCDB is to only record the dose to the primary tumor when an SIB regimen is employed, and suggests potential bias in defining actuarial ENI dose. This is in part, addressed by our implementation of the 37.5–53.1 Gy BED10 dose range, as this would remove SIB regimens employing higher primary tumor doses, but does not address dose levels within our elective range. This suggests potential bias where some ENI doses are recorded as higher than those actually employed.

Lastly, the model was defined in those who went on to receive oncologic surgery. While this represents the minority of patients in PDAC, this was ultimately required given the primary endpoint of ypN disease status. Thus, these findings do not allow us to make conclusions on how our model performs in those unfit for surgery. Rather, this model focuses on the characteristics of those who had successful versus unsuccessful treatment when surgery takes place. Despite these limitations, these findings provide much‐needed context for controversies regarding neoadjuvant therapies in PDAC, while similarly providing the only clinical tool available to quantify treatment response for NAC and radiotherapy with ENI.

## 5 Conclusions

Using a supervised machine learning approach, we describe the first nomogram capable of predicting which patients have a favorable response to NAC and ENI, with higher rates of ypN0 disease, improved R0 resection rates, and longer overall survival. Pending future validation, these findings could be used to identify patients fit to proceed to surgery after neoadjuvant treatment, while similarly designating others for escalation of therapy.

## Author Contributions

**Garrett K. Harada:** conceptualization (lead), data curation (lead), formal analysis (lead), investigation (lead), methodology (lead), project administration (equal), resources (equal), software (equal), supervision (equal), validation (equal), visualization (equal), writing – original draft (lead), writing – review and editing (lead). **Jino Park:** investigation (equal), project administration (equal), resources (equal), validation (equal), writing – original draft (equal), writing – review and editing (equal). **Nicholas Peterson:** investigation (equal), project administration (equal), resources (equal), validation (equal), writing – original draft (equal), writing – review and editing (equal). **Akul Munjal:** investigation (equal), project administration (equal), resources (equal), validation (equal), writing – original draft (equal), writing – review and editing (equal). **David Imagawa:** investigation (equal), project administration (equal), resources (equal), supervision (equal), validation (equal), writing – original draft (equal), writing – review and editing (equal). **Zeljka Jutric:** investigation (equal), project administration (equal), resources (equal), supervision (equal), validation (equal), writing – original draft (equal), writing – review and editing (equal). **Farshid Dayyani:** investigation (equal), project administration (equal), resources (equal), supervision (equal), validation (equal), writing – original draft (equal), writing – review and editing (equal). **Jennifer Valerin:** investigation (equal), project administration (equal), resources (equal), supervision (equal), validation (equal), writing – original draft (equal), writing – review and editing (equal). **David S. Hong:** investigation (equal), project administration (equal), resources (equal), supervision (equal), validation (equal), writing – original draft (equal), writing – review and editing (equal). **Steven N. Seyedin:** conceptualization (equal), data curation (equal), formal analysis (equal), investigation (equal), methodology (equal), project administration (equal), resources (equal), software (equal), supervision (equal), validation (equal), visualization (equal), writing – original draft (equal), writing – review and editing (equal).

## Funding

The authors have nothing to report.

## Ethics Statement

The authors are accountable for all aspects of the work in ensuring that questions related to the accuracy or integrity of any part of the work are appropriately investigated and resolved.

## Conflicts of Interest

**F.D**.: Honoraria: Astellas, AstraZeneca, Eisai, Exelixis, Ipsen, Servier, Sirtex, Takeda; research grant to institution: Amgen, Astellas, AstraZeneca, Bayer, Eisai, Exelixis, Ipsen, Roche, Signatera. **J.V**.: Honoraria: AstraZeneca. The other authors have no conflicts of interest to declare.

## Supporting information


**Figure S1:** Receiver operating characteristics curves and calibration plots—training and testing cohorts. Model receiver operative characteristics curves (Left) and calibration plots (Right) are displayed for training (Top) and testing (Bottom) cohorts, respectively. Model calibration plots are assessed using a fractional polynomial logistic regression model to generate a curve relating the observed versus expected probabilities for ypN0 after ENI and associated 95% confidence intervals. ROC, receiver operative characteristics.

**Figure S2:** Kaplan–Meier and cumulative incidence function survival analysis—ENI‐Naïve Cohort. Kaplan–Meier (Top) and cumulative incidence survival (Bottom) curves are displayed for the ENI‐naïve cohort. All patients were observed beginning at the date of surgery and censored to the date of final follow‐up or death. *p* values for the log‐rank test are displayed for Kaplan–Meier survival estimates whereas subdistribution hazard ratios, 95% confidence intervals, and Gray's test *p* values are shown for cumulative incidence curves. Competing risks were defined as 90‐day postoperative mortality. The threshold for statistical significance was set to *p* < 0.05. BED10, biologically effective dose using an α/β = 10 Gy; ENI, elective nodal irradiation.

**Table S1:** Outcomes by predicted ypN status after neoadjuvant chemotherapy and radiotherapy with ENI—training and testing cohorts. Confusion matrices for assessed outcomes are displayed relative to predicted ypN status. Comparisons between cohorts are performed using chi‐square. Bolded values indicate statistical significance at *p* < 0.05.

**Table S2:** Baseline patient, treatment, and tumor characteristics by receipt of elective nodal irradiation. Baseline demographic, treatment, and tumor characteristics are displayed by receipt of ENI and for the combined cohort. Comparisons between groups are performed using a combination of chi‐square and Wilcoxon rank sum for categorical and continuous variables, respectively. Bolded values indicate statistical significance at *p* < 0.05. 3DCRT, 3‐D conformal radiation therapy; BED10, biologically effective dose using an α/β = 10 Gy; IMRT, intensity modulated radiation therapy; IQR, interquartile range; NAC, neoadjuvant chemotherapy; NOS, not otherwise specified.

**File S1:** Missing data assessment for model covariates and patterns of missingness.

**File S2:** Case Vignette with example nomogram use.

## Data Availability

All data herein was derived directly from the National Cancer Database (NCDB).
