# Supplementary material for: Predicting Benefit of Neoadjuvant Chemotherapy and Elective Nodal Irradiation in Pancreatic Adenocarcinoma: A Supervised Machine Learning Approach
Source: Cancer Med. 2025 Dec 5;14(23):e71447. doi: 10.1002/cam4.71447 (PMC12679486; doi:10.1002/cam4.71447)

**Appendix Figure 1. Receiver Operating Characteristics Curves & Calibration Plots – Training and Testing Cohorts**


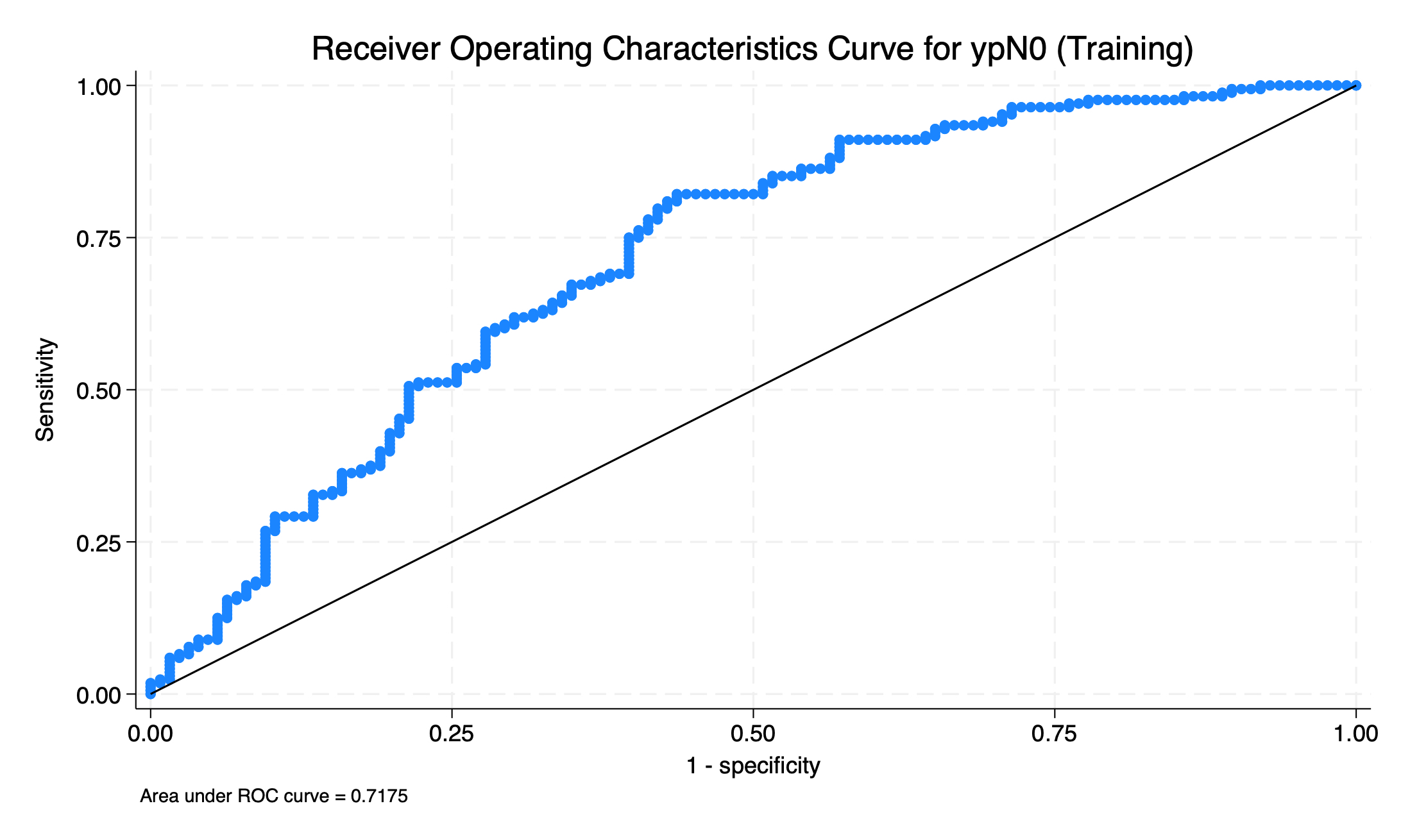

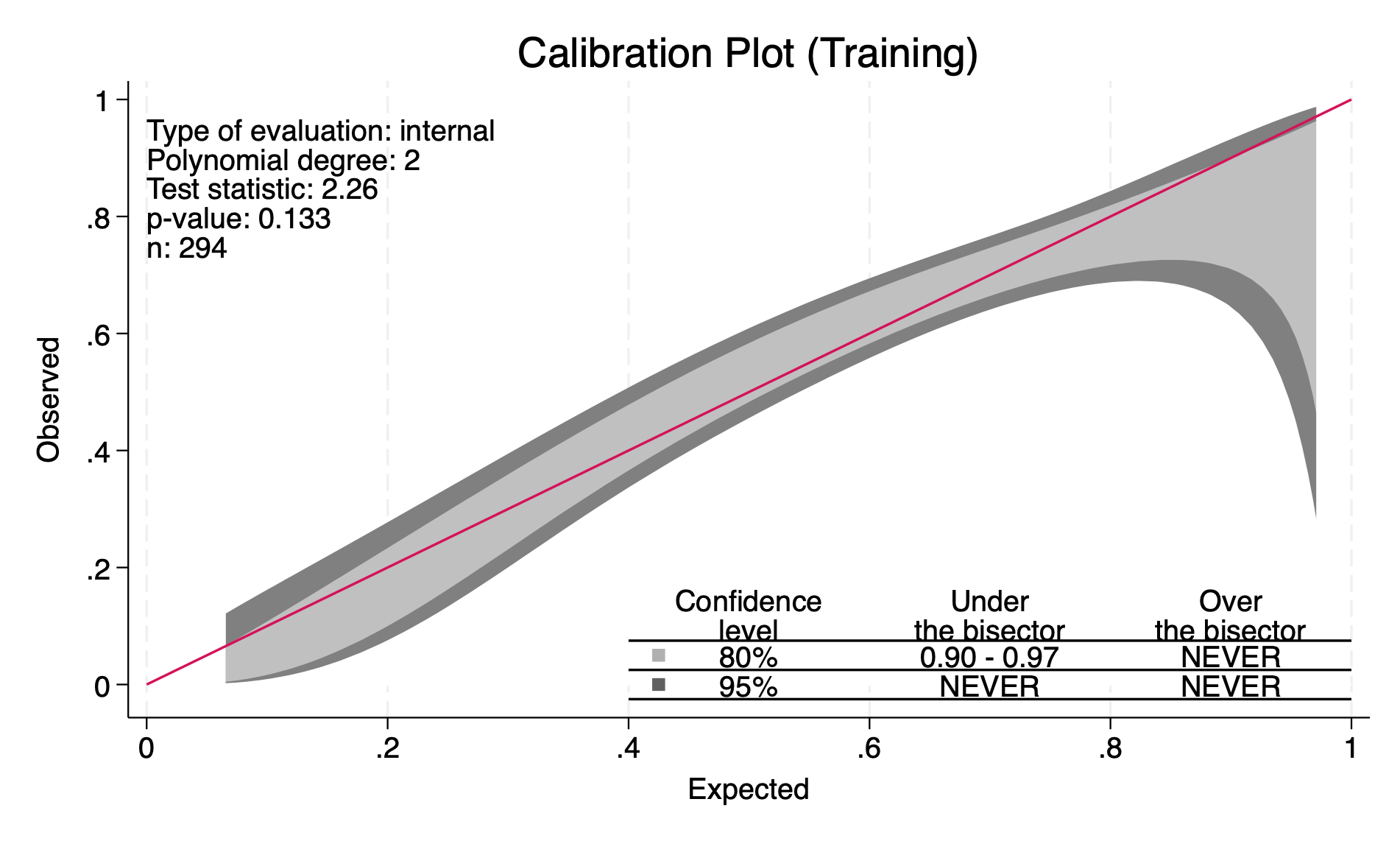


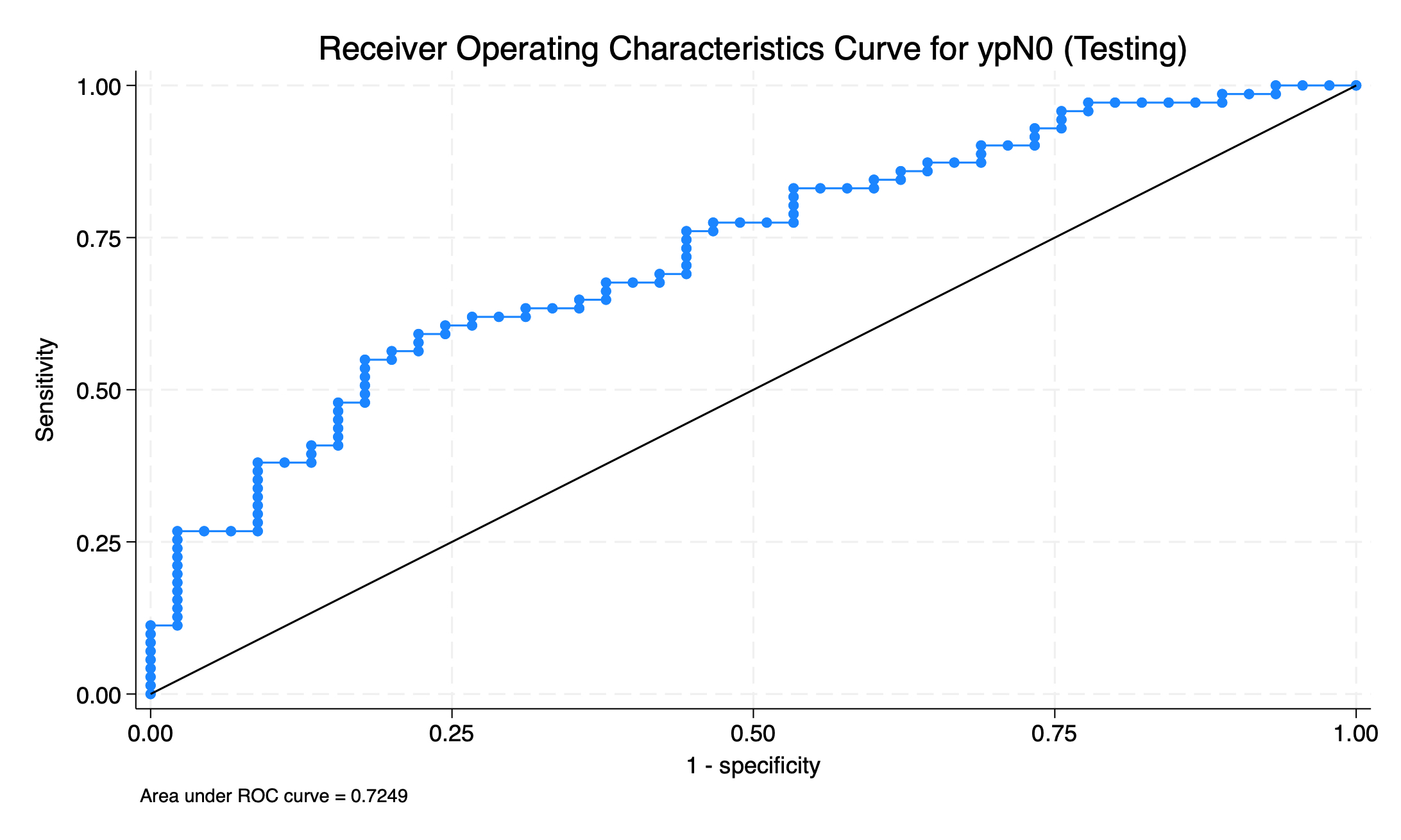

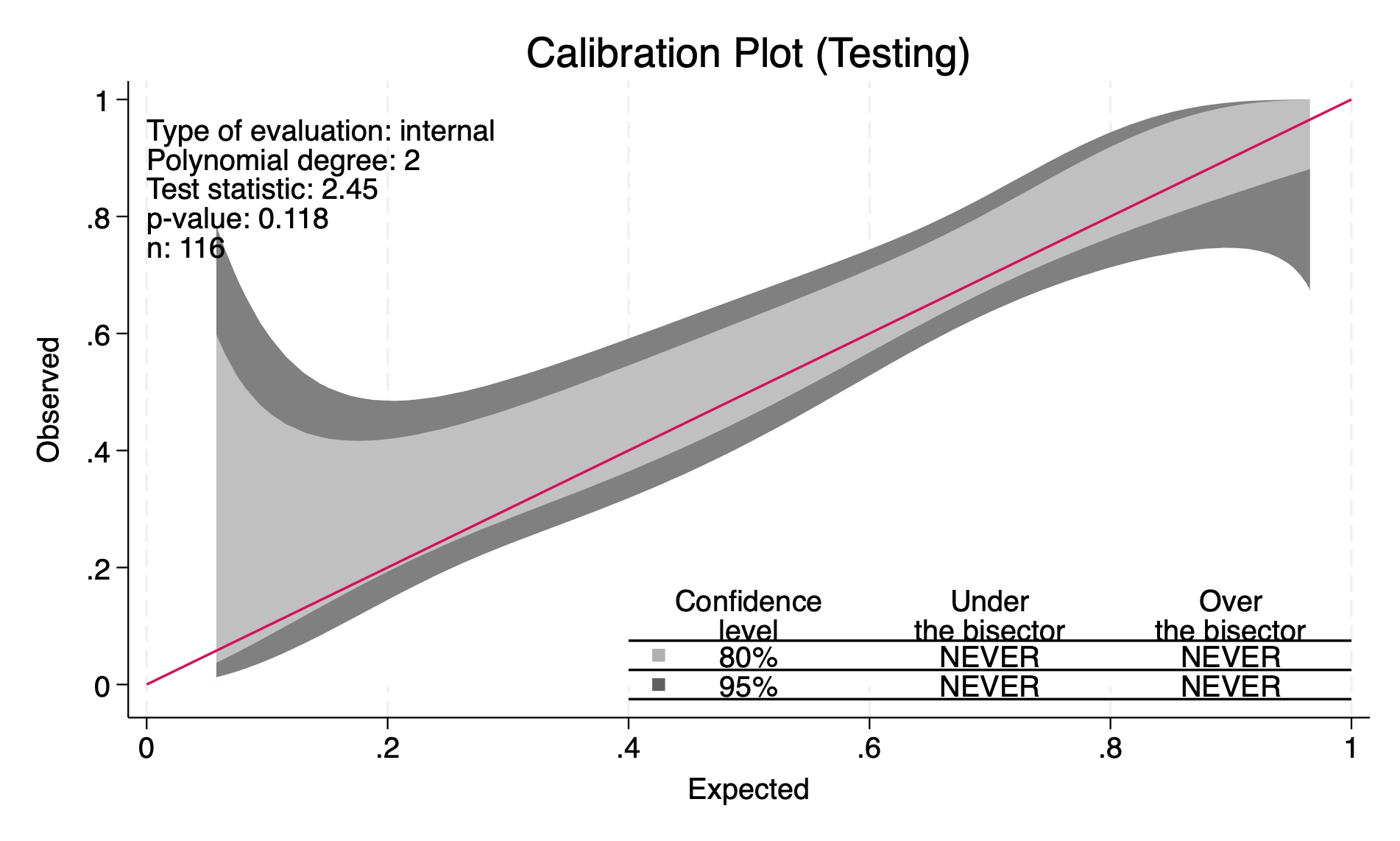

Supplement: Supplementary file 1 — Figure S1: Receiver operating characteristics curves and calibration plots—training and testing cohorts. Model receiver operative characteristics curves (Left) and calibration plots (Right) are displayed for training (Top) and testing (Bottom) cohorts, respectively. Model calibration plots are assessed using a fractional polynomial logistic regression model to generate a curve relating the observed versus expected probabilities for ypN0 after ENI and associated 95% confidence intervals. ROC, receiver operative characteristics. [file CAM4-14-e71447-s003.docx]
