# Supplementary material for: Predicting Benefit of Neoadjuvant Chemotherapy and Elective Nodal Irradiation in Pancreatic Adenocarcinoma: A Supervised Machine Learning Approach
Source: Cancer Med. 2025 Dec 5;14(23):e71447. doi: 10.1002/cam4.71447 (PMC12679486; doi:10.1002/cam4.71447)

**Appendix Figure 2. Kaplan-Meier & Cumulative Incidence Function Survival Analysis – ENI-Naïve Cohort**


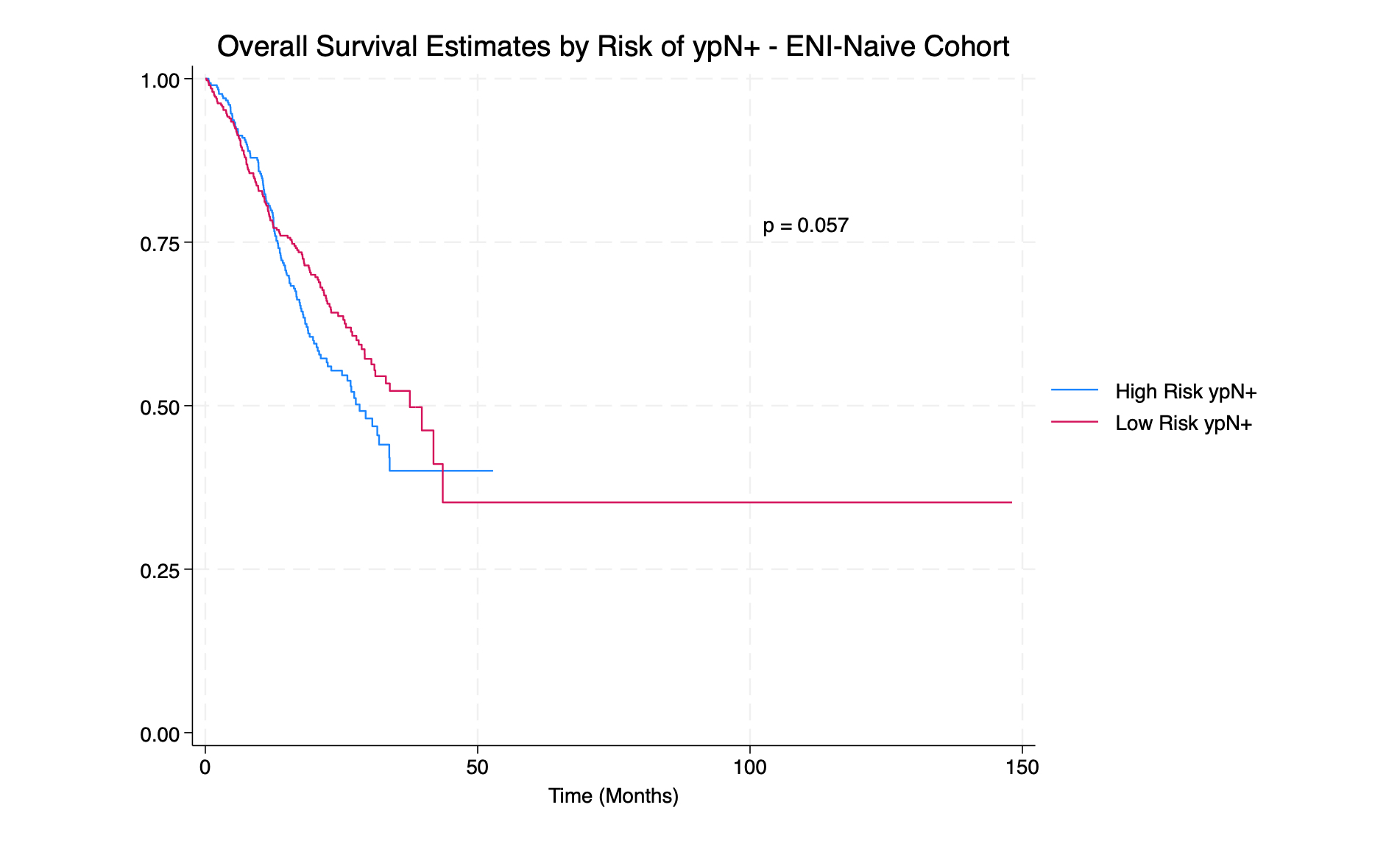


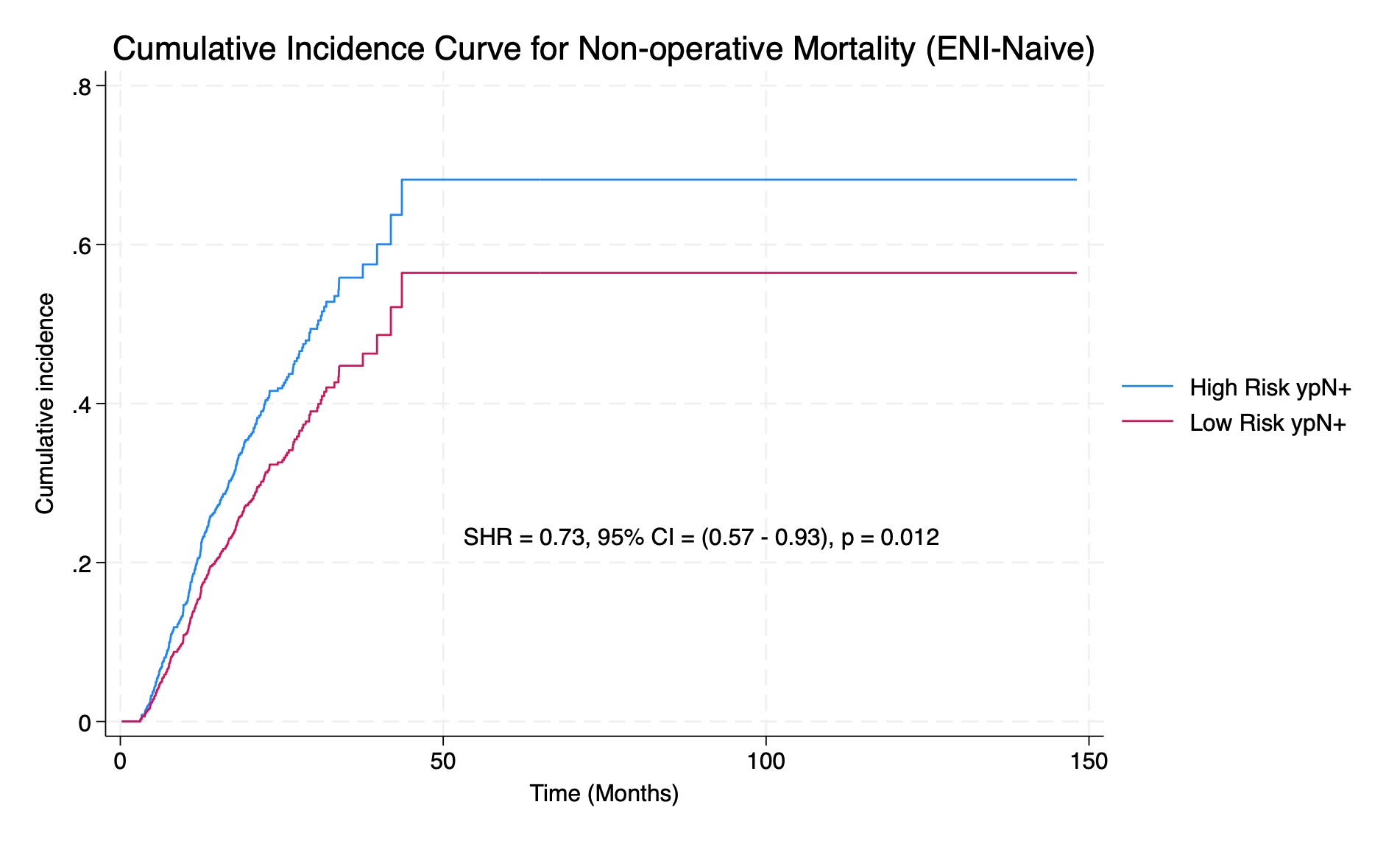

Supplement: Supplementary file 2 — Figure S2: Kaplan–Meier and cumulative incidence function survival analysis—ENI‐Naïve Cohort. Kaplan–Meier (Top) and cumulative incidence survival (Bottom) curves are displayed for the ENI‐naïve cohort. All patients were observed beginning at the date of surgery and censored to the date of final follow‐up or death. p values for the log‐rank test are displayed for Kaplan–Meier survival estimates whereas subdistribution hazard ratios, 95% confidence intervals, and Gray's test p values are shown for cumulative incidence curves. Competing risks were defined as 90‐day postoperative mortality. The threshold for statistical significance was set to p < 0.05. BED10, biologically effective dose using an α/β = 10 Gy; ENI, elective nodal irradiation. [file CAM4-14-e71447-s001.docx]
