# Supplementary material for: Predicting Benefit of Neoadjuvant Chemotherapy and Elective Nodal Irradiation in Pancreatic Adenocarcinoma: A Supervised Machine Learning Approach
Source: Cancer Med. 2025 Dec 5;14(23):e71447. doi: 10.1002/cam4.71447 (PMC12679486; doi:10.1002/cam4.71447)
Supplement: Supplementary file 3 — Table S1: Outcomes by predicted ypN status after neoadjuvant chemotherapy and radiotherapy with ENI—training and testing cohorts. Confusion matrices for assessed outcomes are displayed relative to predicted ypN status. Comparisons between cohorts are performed using chi‐square. Bolded values indicate statistical significance at p < 0.05. [file CAM4-14-e71447-s006.docx]

**Appendix Table 1. Outcomes by Predicted ypN Status after Neoadjuvant Chemotherapy and Radiotherapy with ENI – Training & Testing Cohorts**

|  | Training Cohort | | | |
| --- | --- | --- | --- | --- |
|  | Predicted ypN+ | Predicted ypN0 | Total | p-value |
| N | 101 (34.4%) | 193 (65.6%) | 294 (100.0%) |  |
| ypN Status |  |  |  |  |
| ypN0 | 30 (29.7%) | 138 (71.5%) | 168 (57.1%) | **<0.001** |
| ypN+ | 71 (70.3%) | 55 (28.5%) | 126 (42.9%) |  |
| Tumor Primary Response |  |  |  |  |
| No Response | 7 (22.6%) | 22 (22.4%) | 29 (22.5%) | 0.988 |
| Stable to Partial Response | 24 (77.4%) | 76 (77.6%) | 100 (77.5%) |  |
| Margin Status |  |  |  |  |
| Positive | 37 (37.4%) | 24 (12.7%) | 61 (21.2%) | **<0.001** |
| Negative | 62 (62.6%) | 165 (87.3%) | 227 (78.8%) |  |
|  | Testing Cohort | | | |
|  | Predicted ypN+ | Predicted ypN0 | Total | p-value |
| N | 45 (38.8%) | 71 (61.2%) | 116 (100.0%) |  |
| ypN status |  |  |  |  |
| ypN0 | 20 (44.4%) | 51 (71.8%) | 71 (61.2%) | **0.003** |
| ypN+ | 25 (55.6%) | 20 (28.2%) | 45 (38.8%) |  |
| Tumor Primary Response |  |  |  |  |
| No Response | 2 (10.5%) | 10 (24.4%) | 12 (20.0%) | 0.212 |
| Stable to Partial Response | 17 (89.5%) | 31 (75.6%) | 48 (80.0%) |  |
| Margin Status |  |  |  |  |
| Positive | 18 (40.0%) | 7 (10.1%) | 25 (21.9%) | **<0.001** |
| Negative | 27 (60.0%) | 62 (89.9%) | 89 (78.1%) |  |

Confusion matrices for assessed outcomes are displayed relative to predicted ypN status. Comparisons between cohorts are performed using chi-square. Bolded values indicate statistical significance at p < 0.05.
