# Supplementary material for: Predicting Benefit of Neoadjuvant Chemotherapy and Elective Nodal Irradiation in Pancreatic Adenocarcinoma: A Supervised Machine Learning Approach
Source: Cancer Med. 2025 Dec 5;14(23):e71447. doi: 10.1002/cam4.71447 (PMC12679486; doi:10.1002/cam4.71447)
Supplement: Supplementary file 4 — Table S2: Baseline patient, treatment, and tumor characteristics by receipt of elective nodal irradiation. Baseline demographic, treatment, and tumor characteristics are displayed by receipt of ENI and for the combined cohort. Comparisons between groups are performed using a combination of chi‐square and Wilcoxon rank sum for categorical and continuous variables, respectively. Bolded values indicate statistical significance at p < 0.05. 3DCRT, 3‐D conformal radiation therapy; BED10, biologically effective dose using an α/β = 10 Gy; IMRT, intensity modulated radiation therapy; IQR, interquartile range; NAC, neoadjuvant chemotherapy; NOS, not otherwise specified. [file CAM4-14-e71447-s002.docx]

**Appendix Table 2. Baseline Patient, Treatment, & Tumor Characteristics by Receipt of Elective Nodal Irradiation**

|  | ENI-naïve | ENI | Total | p-value |
| --- | --- | --- | --- | --- |
| N | 1,258 (54.4%) | 1,053 (45.6%) | 2,311 (100.0%) |  |
| Age (Years) [IQR] | 65.0 [58.0 - 71.0] | 64.0 [57.0 - 70.0] | 64.0 [58.0 - 70.0] | **<0.001** |
| Sex |  |  |  |  |
| Male | 614 (48.8%) | 524 (49.8%) | 1,138 (49.2%) | 0.647 |
| Female | 644 (51.2%) | 529 (50.2%) | 1,173 (50.8%) |  |
| Race |  |  |  |  |
| American Indian | 3 (0.2%) | 4 (0.4%) | 7 (0.3%) | 0.151 |
| Asian Indian | 3 (0.2%) | 3 (0.3%) | 6 (0.3%) |  |
| Asian Indian or Pakistani, NOS | 0 (0.0%) | 2 (0.2%) | 2 (0.1%) |  |
| Black | 118 (9.5%) | 99 (9.5%) | 217 (9.5%) |  |
| Chinese | 7 (0.6%) | 3 (0.3%) | 10 (0.4%) |  |
| Filipino | 8 (0.6%) | 0 (0.0%) | 8 (0.3%) |  |
| Japanese | 4 (0.3%) | 1 (0.1%) | 5 (0.2%) |  |
| Korean | 5 (0.4%) | 4 (0.4%) | 9 (0.4%) |  |
| Laotian | 1 (0.1%) | 0 (0.0%) | 1 (0.0%) |  |
| Other | 18 (1.4%) | 6 (0.6%) | 24 (1.0%) |  |
| Other Asian, NOS | 13 (1.0%) | 10 (1.0%) | 23 (1.0%) |  |
| Vietnamese | 2 (0.2%) | 2 (0.2%) | 4 (0.2%) |  |
| White | 1,065 (85.4%) | 907 (87.1%) | 1,972 (86.2%) |  |
| Insurance |  |  |  |  |
| Uninsured | 64 (5.1%) | 67 (6.5%) | 131 (5.7%) | 0.287 |
| Private Insurance / Managed Care | 607 (48.7%) | 468 (45.1%) | 1,075 (47.1%) |  |
| Medicaid | 17 (1.4%) | 11 (1.1%) | 28 (1.2%) |  |
| Medicare | 24 (1.9%) | 17 (1.6%) | 41 (1.8%) |  |
| Other Government | 534 (42.9%) | 474 (45.7%) | 1,008 (44.2%) |  |
| Year of Diagnosis |  |  |  |  |
| 2006 | 6 (0.5%) | 19 (1.8%) | 25 (1.1%) | **<0.001** |
| 2007 | 4 (0.3%) | 17 (1.6%) | 21 (0.9%) |  |
| 2008 | 2 (0.2%) | 27 (2.6%) | 29 (1.3%) |  |
| 2009 | 11 (0.9%) | 31 (2.9%) | 42 (1.8%) |  |
| 2010 | 1 (0.1%) | 31 (2.9%) | 32 (1.4%) |  |
| 2011 | 3 (0.2%) | 51 (4.8%) | 54 (2.3%) |  |
| 2012 | 3 (0.2%) | 61 (5.8%) | 64 (2.8%) |  |
| 2013 | 3 (0.2%) | 66 (6.3%) | 69 (3.0%) |  |
| 2014 | 4 (0.3%) | 98 (9.3%) | 102 (4.4%) |  |
| 2015 | 10 (0.8%) | 152 (14.4%) | 162 (7.0%) |  |
| 2016 | 13 (1.0%) | 171 (16.2%) | 184 (8.0%) |  |
| 2017 | 38 (3.0%) | 181 (17.2%) | 219 (9.5%) |  |
| 2018 | 378 (30.0%) | 60 (5.7%) | 438 (19.0%) |  |
| 2019 | 439 (34.9%) | 50 (4.7%) | 489 (21.2%) |  |
| 2020 | 343 (27.3%) | 38 (3.6%) | 381 (16.5%) |  |
| Treatment Facility Type |  |  |  |  |
| Community Cancer Program | 28 (2.3%) | 26 (2.5%) | 54 (2.4%) | 0.328 |
| Comprehensive Community Cancer Program | 213 (17.1%) | 206 (19.8%) | 419 (18.3%) |  |
| Academic/Research Program | 741 (59.6%) | 586 (56.2%) | 1,327 (58.0%) |  |
| Integrated Network Cancer Program | 262 (21.1%) | 224 (21.5%) | 486 (21.3%) |  |
| Baseline CA 19-9 (U/mL) [IQR] | 980.0 [245.0 - 980.0] | 980.0 [302.0 - 980.0] | 980.0 [295.0 - 980.0] | 0.413 |
| Tumor Primary Location |  |  |  |  |
| Head | 909 (72.3%) | 809 (76.8%) | 1,718 (74.3%) | **0.046** |
| Body | 163 (13.0%) | 101 (9.6%) | 264 (11.4%) |  |
| Tail | 61 (4.8%) | 37 (3.5%) | 98 (4.2%) |  |
| Other | 25 (2.0%) | 23 (2.2%) | 48 (2.1%) |  |
| Overlapping | 55 (4.4%) | 53 (5.0%) | 108 (4.7%) |  |
| Unspecified | 45 (3.6%) | 30 (2.8%) | 75 (3.2%) |  |
| Grade |  |  |  |  |
| 1 | 6 (11.1%) | 61 (12.4%) | 67 (12.3%) | 0.882 |
| 2 | 30 (55.6%) | 268 (54.7%) | 298 (54.8%) |  |
| 3 | 18 (33.3%) | 156 (31.8%) | 174 (32.0%) |  |
| 4 | 0 (0.0%) | 5 (1.0%) | 5 (0.9%) |  |
| AJCC TNM Edition |  |  |  |  |
| 6th Edition | 23 (1.8%) | 94 (8.9%) | 117 (5.1%) | **<0.001** |
| 7th Edition | 75 (6.0%) | 811 (77.0%) | 886 (38.3%) |  |
| 8th Edition | 1,160 (92.2%) | 148 (14.1%) | 1,308 (56.6%) |  |
| Clinical T Stage |  |  |  |  |
| cT1 | 116 (9.2%) | 57 (5.4%) | 173 (7.5%) | **<0.001** |
| cT2 | 639 (50.9%) | 309 (29.4%) | 948 (41.1%) |  |
| cT3 | 232 (18.5%) | 471 (44.8%) | 703 (30.5%) |  |
| cT4 | 269 (21.4%) | 215 (20.4%) | 484 (21.0%) |  |
| Time from Diagnosis to Systemic Therapy (Days) [IQR] | 26.0 [19.0 - 35.0] | 27.0 [19.0 - 37.0] | 27.0 [19.0 - 36.0] | 0.413 |
| Weeks of NAC Delivered |  |  |  |  |
| ≤ 4 | 2 (0.2%) | 16 (1.5%) | 18 (0.8%) | **<0.001** |
| 5 – 8 | 9 (0.7%) | 5 (0.5%) | 14 (0.6%) |  |
| 9 – 12 | 48 (3.8%) | 87 (8.3%) | 135 (5.9%) |  |
| 13 – 16 | 89 (7.1%) | 127 (12.1%) | 216 (9.4%) |  |
| 17 – 20 | 136 (10.8%) | 151 (14.4%) | 287 (12.5%) |  |
| ≥ 21 | 972 (77.4%) | 662 (63.2%) | 1,634 (70.9%) |  |
| Time from Diagnosis to Radiation Therapy (Days) [IQR] | 165.0 [133.0 - 214.0] | 146.0 [110.0 - 200.0] | 159.0 [122.0 - 209.0] | **<0.001** |
| Radiation Technique |  |  |  |  |
| 3DCRT | 93 (7.4%) | 105 (10.0%) | 198 (8.6%) | **0.027** |
| IMRT | 579 (46.0%) | 369 (35.0%) | 948 (41.0%) | **<0.001** |
| Duration of Radiation Therapy (Days) [IQR] | 33.0 [11.0 - 39.0] | 38.0 [24.0 - 41.0] | 36.0 [14.0 - 40.0] | **<0.001** |
| BED10 Delivered to Primary Tumor (Gy) [IQR] | 60.0 [54.8 - 63.7] | 53.1 [48.0 - 59.5] | 59.5 [52.23 - 63.7] | **<0.001** |
| Time from Diagnosis to Definitive Surgery (Days) [IQR] | 210.0 [172.0 - 249.5] | 189.0 [147.0 - 233.0] | 202.0 [160.0 - 244.0] | **<0.001** |
| Pathologic Tumor Size (mm) [IQR] | 31.0 [25.0 - 40.0] | 32.0 [25.0 - 40.0] | 31.0 [25.0 - 40.0] | 0.427 |
| Number of Lymph Nodes Metastatically Involved [IQR] | 0.0 [0.0 - 1.0] | 0.0 [0.0 - 2.0] | 0.0 [0.0 - 2.0] | **0.025** |
| Number of Lymph Nodes Dissected [IQR] | 19.0 [14.0 - 26.0] | 16.0 [11.0 - 23.0] | 18.0 [13.0 - 24.0] | **<0.001** |
| Surgery Type |  |  |  |  |
| Partial Pancreatectomy | 170 (13.5%) | 120 (11.4%) | 290 (12.5%) | 0.575 |
| Partial Pancreatectomy & Duodenectomy | 94 (7.5%) | 70 (6.6%) | 164 (7.1%) |  |
| Partial Pancreatectomy & Duodenectomy without Distal/Partial Gastrectomy | 102 (8.1%) | 98 (9.3%) | 200 (8.7%) |  |
| Partial Pancreatectomy & Duodenectomy with Distal/Partial Gastrectomy (Whipple) | 674 (53.6%) | 563 (53.5%) | 1,237 (53.5%) |  |
| Total Pancreatectomy | 44 (3.5%) | 38 (3.6%) | 82 (3.5%) |  |
| Total Pancreatectomy and Subtotal Gastrectomy | 104 (8.3%) | 98 (9.3%) | 202 (8.7%) |  |
| Extended Pancreatoduodenectomy | 70 (5.6%) | 66 (6.3%) | 136 (5.9%) |  |
| Margin Status |  |  |  |  |
| Positive | 238 (19.3%) | 211 (20.5%) | 449 (19.8%) | 0.472 |
| Negative | 998 (80.7%) | 820 (79.5%) | 1,818 (80.2%) |  |
| Pathologic T Stage |  |  |  |  |
| pT0 | 6 (3.4%) | 33 (3.8%) | 39 (3.7%) | **<0.001** |
| pT1 | 14 (8.0%) | 142 (16.2%) | 156 (14.8%) |  |
| pT1a | 4 (2.3%) | 2 (0.2%) | 6 (0.6%) |  |
| pT1b | 5 (2.8%) | 2 (0.2%) | 7 (0.7%) |  |
| pT1c | 12 (6.8%) | 3 (0.3%) | 15 (1.4%) |  |
| pT2 | 55 (31.2%) | 134 (15.3%) | 189 (18.0%) |  |
| pT3 | 70 (39.8%) | 532 (60.8%) | 602 (57.3%) |  |
| pT4 | 10 (5.7%) | 26 (3.0%) | 36 (3.4%) |  |
| pTis | 0 (0.0%) | 1 (0.1%) | 1 (0.1%) |  |
| Pathologic N Stage |  |  |  |  |
| pN0 | 771 (61.3%) | 600 (57.0%) | 1,371 (59.3%) | **<0.001** |
| pN1 | 373 (29.7%) | 431 (40.9%) | 804 (34.8%) |  |
| pN2 | 114 (9.1%) | 22 (2.1%) | 136 (5.9%) |  |
| Pathologic M Stage |  |  |  |  |
| pM0 | 156 (98.7%) | 451 (99.8%) | 607 (99.5%) | 0.106 |
| pM1 | 2 (1.3%) | 1 (0.2%) | 3 (0.5%) |  |
| Lymphovascular Invasion | 315 (36.0%) | 246 (35.4%) | 561 (35.7%) | 0.804 |
| Primary Tumor Treatment Response | 121 (77.6%) | 355 (79.1%) | 476 (78.7%) | 0.693 |

Baseline demographic, treatment, and tumor characteristics are displayed by receipt of ENI and for the combined cohort. Comparisons between groups are performed using a combination of chi-square and Wilcoxon rank sum for categorical and continuous variables, respectively. Bolded values indicate statistical significance at p < 0.05.

IQR = Interquartile range; NAC = Neoadjuvant chemotherapy; 3DCRT = 3-D conformal radiation therapy; IMRT = Intensity modulated radiation therapy; BED10 = Biologically effective dose using an 𝛂/𝛃= 10 Gy; NOS = Not otherwise specified.
