# Supplementary material for: Predicting Benefit of Neoadjuvant Chemotherapy and Elective Nodal Irradiation in Pancreatic Adenocarcinoma: A Supervised Machine Learning Approach
Source: Cancer Med. 2025 Dec 5;14(23):e71447. doi: 10.1002/cam4.71447 (PMC12679486; doi:10.1002/cam4.71447)
Supplement: Supplementary file 5 — File S1: Missing data assessment for model covariates and patterns of missingness. [file CAM4-14-e71447-s005.docx]

| Supplementary File 1A. Missing Data by Variable for Training Data | |  |  |
| --- | --- | --- | --- |
| Variable | Missing Observations | Complete Observations | % Missing |
| Age | 0 | 1053 | 0.0 |
| Sex | 0 | 1053 | 0.0 |
| Race | 12 | 1041 | 1.1 |
| Insurance | 16 | 1037 | 1.5 |
| Crowfly | 128 | 925 | 12.2 |
| Charlson-Deyo Score | 0 | 1053 | 0.0 |
| Facility Type | 11 | 1042 | 1.0 |
| Year of Diagnosis | 0 | 1053 | 0.0 |
| AJCC Edition Number | 0 | 1053 | 0.0 |
| Clinical T-Stage | 0 | 1053 | 0.0 |
| Histologic Grade | 563 | 490 | 53.5 |
| CA 19-9 | 518 | 535 | 49.2 |
| Primary Site | 0 | 1053 | 0.0 |
| CS Extension | 500 | 553 | 47.5 |
| Time from Diagnosis to Systemic Therapy | 49 | 1004 | 4.7 |
| Time from Diagnosis to Radiation Therapy | 60 | 993 | 5.7 |
| Time from Diagnosis to Definitive Surgery | 5 | 1048 | 0.5 |
| Duration of Radiation Therapy | 301 | 752 | 28.6 |
| 3D Conformal | 0 | 1053 | 0.0 |
| IMRT | 0 | 1053 | 0.0 |
| Baseline preoperative variables considered for model inclusion with respective percentages of missing data. A threshold for missing data exclusion was established at the level of missing data for elective nodal irradiation dose (>40%). | | | |
| Crowfly = Straight line distance in miles between the patient's residence and treating facility; CS Extension = Collaborative Stage Extension variable encoding data describing primary tumor invasion into adjacent structures. IMRT = Intensity-modulated radiation therapy. | | | |

**Supplementary File 1. Missing Data Assessment for Model Covariates and Patterns of Missingness**

| Supplementary File 1B. Patterns of Missingness - Training Data | | | | | | | | | | | | |  |
| --- | --- | --- | --- | --- | --- | --- | --- | --- | --- | --- | --- | --- | --- |
| Missing Data Pattern | Percent | Time from Diagnosis to Definitive Surgery | Facility Type | Race | Insurance | Time from Diagnosis to Systemic Therapy | Time from Diagnosis to Radiation Therapy | Crowfly | Duration of Radiation Therapy | CS Extension | CA 19-9 | Histologic Grade |  |
|  |  |  |  |  |  |  |  |  |  |  |  |  |  |
| 1 | 8 | 1 | 1 | 1 | 1 | 1 | 1 | 1 | 1 | 1 | 1 | 1 |  |
| 2 | 14 | 1 | 1 | 1 | 1 | 1 | 1 | 1 | 1 | 0 | 0 | 0 |  |
| 3 | 8 | 1 | 1 | 1 | 1 | 1 | 1 | 1 | 1 | 0 | 1 | 0 |  |
| 4 | 8 | 1 | 1 | 1 | 1 | 1 | 1 | 1 | 1 | 1 | 0 | 1 |  |
| 5 | 7 | 1 | 1 | 1 | 1 | 1 | 1 | 1 | 1 | 1 | 1 | 0 |  |
| 6 | 5 | 1 | 1 | 1 | 1 | 1 | 1 | 1 | 1 | 0 | 1 | 1 |  |
| 7 | 5 | 1 | 1 | 1 | 1 | 1 | 1 | 1 | 0 | 1 | 1 | 1 |  |
| 8 | 5 | 1 | 1 | 1 | 1 | 1 | 1 | 1 | 1 | 1 | 0 | 0 |  |
| 9 | 4 | 1 | 1 | 1 | 1 | 1 | 1 | 1 | 0 | 1 | 0 | 1 |  |
| 10 | 3 | 1 | 1 | 1 | 1 | 1 | 1 | 0 | 1 | 0 | 0 | 0 |  |
| 11 | 3 | 1 | 1 | 1 | 1 | 1 | 1 | 1 | 1 | 0 | 0 | 1 |  |
| 12 | 3 | 1 | 1 | 1 | 1 | 1 | 1 | 1 | 0 | 1 | 1 | 0 |  |
| 13 | 2 | 1 | 1 | 1 | 1 | 1 | 1 | 1 | 0 | 0 | 0 | 0 |  |
| 14 | 2 | 1 | 1 | 1 | 1 | 1 | 1 | 1 | 0 | 0 | 1 | 1 |  |
| 15 | 2 | 1 | 1 | 1 | 1 | 1 | 1 | 1 | 0 | 1 | 0 | 0 |  |
| 16 | 2 | 1 | 1 | 1 | 1 | 1 | 1 | 0 | 1 | 0 | 1 | 0 |  |
| 17 | 1 | 1 | 1 | 1 | 1 | 1 | 1 | 1 | 0 | 0 | 1 | 0 |  |
| 18 | 1 | 1 | 1 | 1 | 1 | 1 | 1 | 0 | 1 | 1 | 1 | 1 |  |
| 19 | 1 | 1 | 1 | 1 | 1 | 1 | 1 | 0 | 1 | 1 | 0 | 0 |  |
| 20 | 1 | 1 | 1 | 1 | 1 | 1 | 1 | 1 | 0 | 0 | 0 | 1 |  |
| 21 | <1 | 1 | 1 | 1 | 1 | 1 | 0 | 1 | 0 | 1 | 1 | 1 |  |
| 22 | <1 | 1 | 1 | 1 | 1 | 1 | 1 | 0 | 1 | 0 | 1 | 1 |  |
| 23 | <1 | 1 | 1 | 1 | 1 | 1 | 1 | 0 | 1 | 1 | 0 | 1 |  |
| 24 | <1 | 1 | 1 | 1 | 1 | 1 | 1 | 0 | 1 | 1 | 1 | 0 |  |
| 25 | <1 | 1 | 1 | 1 | 1 | 0 | 0 | 1 | 0 | 1 | 0 | 1 |  |
| 26 | <1 | 1 | 1 | 1 | 1 | 0 | 0 | 1 | 0 | 1 | 1 | 1 |  |
| 27 | <1 | 1 | 1 | 1 | 1 | 1 | 0 | 1 | 0 | 0 | 1 | 1 |  |
| 28 | <1 | 1 | 1 | 1 | 1 | 1 | 0 | 1 | 0 | 1 | 1 | 0 |  |
| 29 | <1 | 1 | 1 | 1 | 1 | 1 | 1 | 0 | 1 | 0 | 0 | 1 |  |
| 30 | <1 | 1 | 1 | 1 | 1 | 1 | 1 | 0 | 0 | 1 | 1 | 0 |  |
| 31 | <1 | 1 | 1 | 1 | 1 | 1 | 1 | 0 | 0 | 1 | 1 | 1 |  |
| 32 | <1 | 1 | 0 | 1 | 1 | 1 | 1 | 1 | 1 | 1 | 0 | 0 |  |
| 33 | <1 | 1 | 1 | 1 | 0 | 1 | 1 | 1 | 1 | 0 | 1 | 1 |  |
| 34 | <1 | 1 | 1 | 1 | 1 | 0 | 1 | 1 | 1 | 0 | 1 | 1 |  |
| 35 | <1 | 1 | 1 | 1 | 1 | 0 | 1 | 1 | 1 | 1 | 1 | 1 |  |
| 36 | <1 | 1 | 1 | 1 | 1 | 1 | 0 | 1 | 0 | 0 | 0 | 0 |  |
| 37 | <1 | 0 | 1 | 1 | 1 | 0 | 0 | 1 | 0 | 1 | 0 | 1 |  |
| 38 | <1 | 0 | 1 | 1 | 1 | 0 | 0 | 1 | 1 | 1 | 0 | 1 |  |
| 39 | <1 | 1 | 0 | 1 | 1 | 1 | 1 | 1 | 1 | 1 | 1 | 1 |  |
| 40 | <1 | 1 | 1 | 0 | 1 | 1 | 1 | 1 | 1 | 0 | 1 | 1 |  |
| 41 | <1 | 1 | 1 | 1 | 0 | 1 | 1 | 0 | 1 | 0 | 0 | 0 |  |
| 42 | <1 | 1 | 1 | 1 | 0 | 1 | 1 | 1 | 1 | 1 | 1 | 1 |  |
| 43 | <1 | 1 | 1 | 1 | 1 | 0 | 0 | 1 | 0 | 0 | 1 | 1 |  |
| 44 | <1 | 1 | 1 | 1 | 1 | 0 | 0 | 1 | 0 | 1 | 0 | 0 |  |
| 45 | <1 | 1 | 1 | 1 | 1 | 0 | 0 | 1 | 0 | 1 | 1 | 0 |  |
| 46 | <1 | 1 | 1 | 1 | 1 | 0 | 1 | 1 | 1 | 0 | 1 | 0 |  |
| 47 | <1 | 1 | 1 | 1 | 1 | 0 | 1 | 1 | 1 | 1 | 0 | 0 |  |
| 48 | <1 | 1 | 1 | 1 | 1 | 1 | 0 | 1 | 0 | 0 | 1 | 0 |  |
| 49 | <1 | 1 | 1 | 1 | 1 | 1 | 0 | 1 | 0 | 1 | 0 | 0 |  |
| 50 | <1 | 1 | 1 | 1 | 1 | 1 | 1 | 0 | 0 | 0 | 1 | 0 |  |
| 51 | <1 | 1 | 1 | 1 | 1 | 1 | 1 | 1 | 0 | 0 | 0 | 1 |  |
| 52 | <1 | 0 | 1 | 1 | 1 | 0 | 0 | 0 | 0 | 0 | 0 | 1 |  |
| 53 | <1 | 1 | 0 | 1 | 1 | 1 | 1 | 0 | 1 | 1 | 0 | 1 |  |
| 54 | <1 | 1 | 0 | 1 | 1 | 1 | 1 | 1 | 0 | 0 | 1 | 1 |  |
| 55 | <1 | 1 | 0 | 1 | 1 | 1 | 1 | 1 | 0 | 1 | 0 | 1 |  |
| 56 | <1 | 1 | 0 | 1 | 1 | 1 | 1 | 1 | 1 | 0 | 0 | 0 |  |
| 57 | <1 | 1 | 0 | 1 | 1 | 1 | 1 | 1 | 1 | 0 | 1 | 1 |  |
| 58 | <1 | 1 | 0 | 1 | 1 | 1 | 1 | 1 | 1 | 1 | 1 | 0 |  |
| 59 | <1 | 1 | 1 | 0 | 0 | 1 | 1 | 1 | 1 | 0 | 1 | 0 |  |
| 60 | <1 | 1 | 1 | 0 | 1 | 0 | 0 | 1 | 0 | 1 | 0 | 1 |  |
| 61 | <1 | 1 | 1 | 0 | 1 | 1 | 0 | 1 | 0 | 1 | 0 | 0 |  |
| 62 | <1 | 1 | 1 | 0 | 1 | 1 | 1 | 1 | 0 | 0 | 0 | 1 |  |
| 63 | <1 | 1 | 1 | 0 | 1 | 1 | 1 | 1 | 0 | 0 | 1 | 1 |  |
| 64 | <1 | 1 | 1 | 0 | 1 | 1 | 1 | 1 | 0 | 1 | 0 | 0 |  |
| 65 | <1 | 1 | 1 | 0 | 1 | 1 | 1 | 1 | 0 | 1 | 1 | 1 |  |
| 66 | <1 | 1 | 1 | 0 | 1 | 1 | 1 | 1 | 1 | 0 | 1 | 0 |  |
| 67 | <1 | 1 | 1 | 0 | 1 | 1 | 1 | 1 | 1 | 1 | 0 | 1 |  |
| 68 | <1 | 1 | 1 | 0 | 1 | 1 | 1 | 1 | 1 | 1 | 1 | 1 |  |
| 69 | <1 | 1 | 1 | 1 | 0 | 0 | 1 | 1 | 1 | 1 | 0 | 0 |  |
| 70 | <1 | 1 | 1 | 1 | 0 | 1 | 1 | 0 | 1 | 1 | 1 | 0 |  |
| 71 | <1 | 1 | 1 | 1 | 0 | 1 | 1 | 1 | 0 | 0 | 1 | 1 |  |
| 72 | <1 | 1 | 1 | 1 | 0 | 1 | 1 | 1 | 0 | 1 | 0 | 0 |  |
| 73 | <1 | 1 | 1 | 1 | 0 | 1 | 1 | 1 | 0 | 1 | 0 | 1 |  |
| 74 | <1 | 1 | 1 | 1 | 0 | 1 | 1 | 1 | 1 | 0 | 0 | 0 |  |
| 75 | <1 | 1 | 1 | 1 | 0 | 1 | 1 | 1 | 1 | 1 | 0 | 0 |  |
| 76 | <1 | 1 | 1 | 1 | 0 | 1 | 1 | 1 | 1 | 1 | 1 | 0 |  |
| 77 | <1 | 1 | 1 | 1 | 1 | 0 | 0 | 0 | 0 | 0 | 0 | 0 |  |
| 78 | <1 | 1 | 1 | 1 | 1 | 0 | 0 | 1 | 0 | 0 | 0 | 0 |  |
| 79 | <1 | 1 | 1 | 1 | 1 | 0 | 0 | 1 | 0 | 0 | 0 | 1 |  |
| 80 | <1 | 1 | 1 | 1 | 1 | 0 | 0 | 1 | 0 | 0 | 1 | 0 |  |
| 81 | <1 | 1 | 1 | 1 | 1 | 0 | 1 | 0 | 1 | 0 | 0 | 0 |  |
| 82 | <1 | 1 | 1 | 1 | 1 | 0 | 1 | 0 | 1 | 0 | 1 | 0 |  |
| 83 | <1 | 1 | 1 | 1 | 1 | 0 | 1 | 1 | 0 | 0 | 0 | 0 |  |
| 84 | <1 | 1 | 1 | 1 | 1 | 0 | 1 | 1 | 0 | 0 | 0 | 1 |  |
| 85 | <1 | 1 | 1 | 1 | 1 | 0 | 1 | 1 | 0 | 0 | 1 | 0 |  |
| 86 | <1 | 1 | 1 | 1 | 1 | 0 | 1 | 1 | 0 | 1 | 1 | 0 |  |
| 87 | <1 | 1 | 1 | 1 | 1 | 0 | 1 | 1 | 0 | 1 | 1 | 1 |  |
| 88 | <1 | 1 | 1 | 1 | 1 | 0 | 1 | 1 | 1 | 0 | 0 | 0 |  |
| 89 | <1 | 1 | 1 | 1 | 1 | 0 | 1 | 1 | 1 | 0 | 0 | 1 |  |
| 90 | <1 | 1 | 1 | 1 | 1 | 1 | 0 | 0 | 0 | 0 | 0 | 1 |  |
| 91 | <1 | 1 | 1 | 1 | 1 | 1 | 0 | 0 | 0 | 0 | 1 | 1 |  |
| 92 | <1 | 1 | 1 | 1 | 1 | 1 | 0 | 0 | 0 | 1 | 1 | 1 |  |
| 93 | <1 | 1 | 1 | 1 | 1 | 1 | 0 | 1 | 0 | 0 | 0 | 1 |  |
| 94 | <1 | 1 | 1 | 1 | 1 | 1 | 0 | 1 | 0 | 1 | 0 | 1 |  |
| 95 | <1 | 1 | 1 | 1 | 1 | 1 | 1 | 0 | 0 | 0 | 0 | 0 |  |
| 96 | <1 | 1 | 1 | 1 | 1 | 1 | 1 | 0 | 0 | 0 | 0 | 1 |  |
| 97 | <1 | 1 | 1 | 1 | 1 | 1 | 1 | 0 | 0 | 0 | 1 | 1 |  |
| 98 | <1 | 1 | 1 | 1 | 1 | 1 | 1 | 0 | 0 | 1 | 0 | 1 |  |
| Patterns of missing data with 1 and 0 used to represent presence or absence of data for each respective variable. Only baseline variables with presence of any missing data are displayed. Complete data sets are represented by Missing Data Pattern = 1 with subsequent patterns of missing data listed in order of decreasing incidence. Highlighted patterns represent those excluded following removal of CS Extension, CA 19-9, and Histologic Grade for a fraction of missing data >40%. | | | | | | | | | | | | |  |
| Crowfly = Straight line distance in miles between the patient's residence and treating facility; CS Extension = Collaborative Stage Extension variable encoding data describing primary tumor invasion into adjacent structures. | | | | | | | | | | | | |  |

| Supplementary File 1C. Patterns of Missingness After Exclusions for >40% Missing Fraction - Training Data | | | | | | | | | |  |
| --- | --- | --- | --- | --- | --- | --- | --- | --- | --- | --- |
| Missing Data Pattern | Percent | Time from Diagnosis to Definitive Surgery | Facility Type | Race | Insurance | Time from Diagnosis to Systemic Therapy | Time from Diagnosis to Radiation Therapy | Crowfly | Duration of Radiation Therapy |  |
|  |  |  |  |  |  |  |  |  |  |  |
| 1 | 58 | 1 | 1 | 1 | 1 | 1 | 1 | 1 | 1 |  |
| 2 | 20 | 1 | 1 | 1 | 1 | 1 | 1 | 1 | 0 |  |
| 3 | 10 | 1 | 1 | 1 | 1 | 1 | 1 | 0 | 1 |  |
| 4 | 3 | 1 | 1 | 1 | 1 | 1 | 0 | 1 | 0 |  |
| 5 | 2 | 1 | 1 | 1 | 1 | 0 | 0 | 1 | 0 |  |
| 6 | 1 | 1 | 1 | 1 | 1 | 1 | 1 | 0 | 0 |  |
| 7 | 1 | 1 | 1 | 1 | 1 | 0 | 1 | 1 | 1 |  |
| 8 | <1 | 1 | 0 | 1 | 1 | 1 | 1 | 1 | 1 |  |
| 9 | <1 | 1 | 1 | 1 | 0 | 1 | 1 | 1 | 1 |  |
| 10 | <1 | 1 | 1 | 0 | 1 | 1 | 1 | 1 | 1 |  |
| 11 | <1 | 1 | 1 | 1 | 1 | 0 | 1 | 1 | 0 |  |
| 12 | <1 | 1 | 1 | 0 | 1 | 1 | 1 | 1 | 0 |  |
| 13 | <1 | 1 | 1 | 1 | 0 | 1 | 1 | 0 | 1 |  |
| 14 | <1 | 1 | 1 | 1 | 0 | 1 | 1 | 1 | 0 |  |
| 15 | <1 | 1 | 1 | 1 | 1 | 1 | 0 | 0 | 0 |  |
| 16 | <1 | 0 | 1 | 1 | 1 | 0 | 0 | 1 | 0 |  |
| 17 | <1 | 0 | 1 | 1 | 1 | 0 | 0 | 1 | 1 |  |
| 18 | <1 | 1 | 0 | 1 | 1 | 1 | 1 | 1 | 0 |  |
| 19 | <1 | 1 | 1 | 1 | 1 | 0 | 1 | 0 | 1 |  |
| 20 | <1 | 1 | 1 | 1 | 1 | 1 | 1 | 1 | 0 |  |
| 21 | <1 | 0 | 1 | 1 | 1 | 0 | 0 | 0 | 0 |  |
| 22 | <1 | 1 | 0 | 1 | 1 | 1 | 1 | 0 | 1 |  |
| 23 | <1 | 1 | 1 | 0 | 0 | 1 | 1 | 1 | 1 |  |
| 24 | <1 | 1 | 1 | 0 | 1 | 0 | 0 | 1 | 0 |  |
| 25 | <1 | 1 | 1 | 0 | 1 | 1 | 0 | 1 | 0 |  |
| 26 | <1 | 1 | 1 | 1 | 0 | 0 | 1 | 1 | 1 |  |
| 27 | <1 | 1 | 1 | 1 | 1 | 0 | 0 | 0 | 0 |  |
| Patterns of missing data with 1 and 0 used to represent presence or absence of data for each respective variable. Only baseline variables with presence of any missing data are displayed. Complete data sets are represented by Missing Data Pattern = 1 with subsequent patterns of missing data listed in order of decreasing incidence. | | | | | | | | | |  |
| Crowfly = Straight line distance in miles between the patient's residence and treating facility | | | | | | | | | |  |
