# Supplementary material for: Predicting Benefit of Neoadjuvant Chemotherapy and Elective Nodal Irradiation in Pancreatic Adenocarcinoma: A Supervised Machine Learning Approach
Source: Cancer Med. 2025 Dec 5;14(23):e71447. doi: 10.1002/cam4.71447 (PMC12679486; doi:10.1002/cam4.71447)
Supplement: Supplementary file 6 — File S2: Case Vignette with example nomogram use. [file CAM4-14-e71447-s004.docx]

| **Supplementary File 2. Case Vignette with Example Nomogram Use** | | | | | | | | |
| --- | --- | --- | --- | --- | --- | --- | --- | --- |
| Covariate | Minimum Point Value ⱡ | Maximum Point Value ⱡ | Initial Nomogram Score  (Diagnosis) | Updated Nomogram Score (After 7 Cycles FOLFIRINOX) | Updated Nomogram Score (After 10 Cycles FOLFIRINOX) | Updated Nomogram Score (37.5 Gy ENI BED10) | Updated Nomogram Score (53.1 Gy ENI BED10) | Final Nomogram Score (53.1 Gy ENI BED10) |
| Demographics |  |  |  |  |  |  |  |  |
| Charlson-Deyo Score |  |  |  |  |  |  |  |  |
| ≥ 3 | 0 (Score ≥ 3) | 1.2 (Score < 3) | 1.2 | 1.2 | 1.2 | 1.2 | 1.2 | 1.2 |
| 2 | 0 (Score = 2) | 1 (Score ≠ 2) | 1 | 1 | 1 | 1 | 1 | 1 |
| 0 | 0 (Score ≠ 0) | 0.3 (Score = 0) | 0.3 | 0.3 | 0.3 | 0.3 | 0.3 | 0.3 |
| Treating Facility |  |  |  |  |  |  |  |  |
| Integrated Network Cancer Program | 0 (Treated) | 0.4 (Treated Elsewhere) | 0.4 | 0.4 | 0.4 | 0.4 | 0.4 | 0.4 |
| Academic Cancer Program | 0 (Treated Elsewhere) | 2.2 (Treated) | 2.2 | 2.2 | 2.2 | 2.2 | 2.2 | 2.2 |
| Community Cancer Program | 0 (Treated Elsewhere) | 1.7 (Treated) | 0 | 0 | 0 | 0 | 0 | 0 |
| Tumor Features |  |  |  |  |  |  |  |  |
| Pancreatic Body Primary Location | 0 (Head, Tail) | 0.4 (Body) | 0 | 0 | 0 | 0 | 0 | 0 |
| AJCC TNM Staging |  |  |  |  |  |  |  |  |
| 6th Edition | 0 (7th or 8th Edition) | 1.7 (6th Edition) | 0 | 0 | 0 | 0 | 0 | 0 |
| 8th Edition | 0 (8th Edition) | 0.7 (6th or 7th Edition) | 0 | 0 | 0 | 0 | 0 | 0 |
| Treatment Features |  |  |  |  |  |  |  |  |
| ENI BED10 (Gy) | 1.6 (37.5 Gy) | 2.5 (54 Gy) | 0 | 0 | 0 | 1.6 | 2.5 | 2.5 |
| Time from Diagnosis to Radiation (Days) | 1.1 (40 days) | 10 (350 days) | 0 | 3.0 (105 days) | 4.6 (161 days) | 4.6 (168 days) | 4.6 (168 days) | 4.6 (168 days) |
| Time from Diagnosis to Surgery (Days) | 0.4 (70 days) | 1.8 (350 days) | 0 | 0.6 (119 days) | 1.0 (175 days) | 1.0 (187 days) | 1.1 (215 days) | 1.2 (222 days) |
| Treatment Interaction Terms |  |  |  |  |  |  |  |  |
| Weeks of NAC + Radiation Duration (Days) |  |  |  |  |  |  |  |  |
| 9 – 12 Weeks | - 4.7 (80 days) | - 0.3 (5 days) | 0 | 0 | 0 | 0 | 0 | 0 |
| Weeks of NAC + ENI BED10 (Gy) |  |  |  |  |  |  |  |  |
| 9 – 12 Weeks | - 1.5 (54 Gy) | - 1.0 (37.5 Gy) | 0 | 0 | 0 | 0 | 0 | 0 |
| ≥ 21 Weeks | 1.4 (37.5 Gy) | 2.2 (54 Gy) | 0 | 0 | 0 | 1.4 | 2.2 | 2.2 |
| Weeks of NAC + Clinical T Stage |  |  |  |  |  |  |  |  |
| 13 – 16 Weeks + cT1 | 0 (cT2-4) | 1.5 (cT1) | 0 | 0 | 0 | 0 | 0 | 0 |
| 13 – 16 Weeks + cT2 | - 1.4 (cT2) | 0 (cT1, cT3-4) | 0 | -1.4 | 0 | 0 | 0 | 0 |
| 13 – 16 Weeks + cT4 | - 7.6 (cT4) | 0 (cT1-3) | 0 | 0 | 0 | 0 | 0 | 0 |
| 17 – 20 Weeks + cT1 | 0 (cT2-4) | 7.6 (cT1) | 0 | 0 | 0 | 0 | 0 | 0 |
| ≥ 21 Weeks + cT4 | 0 (cT1-3) | 0.4 (cT4) | 0 | 0 | 0 | 0 | 0 | 0 |
| Correction Factor ⱡⱡ | 0.08 | 0.08 | 0.08 | 0.08 | 0.08 | 0.08 | 0.08 | 0.08 |
| **TOTAL** | 0 | 29.9 | 5.2 | 7.4 | 10.8 | 13.2 | 14.0 | 14.1 |
| **PROBABILITY** | < 0.05% | 98.8% | 11.0% | 20.0 % | 36.0% | 51.0% | 56.0% | 56.0% |
| ⱡ Point values represent a scaled measure of the penalized 𝛃 coefficient. Values were calculate using simulated data approximating the distribution of selected covariates within patients randomly assigned to the training cohort. Ranges indicate minimum and maximum values possible given the observed data. Note that where possible, negative coefficients were forced positive with adjustment of all other coefficients in order to facilitate simplicity of calculations. | | | | | | | | |
| ⱡⱡ Given the decision to drop female sex from the model for a negligible effect size, a correction factor was introduced for recalibration. Correction factor accounts for a 0.1 - 2.0% increase in probability of ypN0 after neoadjuvant chemotherapy and radiotherapy with ENI across all cases. | | | | | | | | |
| FOLFIRINOX = 5-fluorouracil, leucovorin, irinotecan, oxaliplatin. Charlson-Deyo Score = Standardized prognostic metric for 10-year survival using a set of comorbid conditions. AJCC TNM Staging = Model adjustments made by American Joint Commission on Cancer staging edition employed due to shifts in both cT and cN criterion from the 6th to 8th editions. Time to Radiation (Days) = Actual range was 40 to 350 days. Time to Surgery (Days) = Actual range was 70 to 350 days. ENI BED10 = Elective nodal irradiation biologically effective dose using an 𝛂/𝛃= 10 Gy. Doses delivered were limited to a BED10 of 37.5 Gy to 54 Gy. NAC = Neoadjuvant chemotherapy. | | | | | | | | |

Here we provide an example scenario illustrating how this nomogram may be employed in clinical practice. Assuming treatment at an academic center, a female patient with no major medical comorbidities (Charlson-Deyo = 0) is diagnosed with a cT2N0M0 (AJCC 8^th^ edition) borderline resectable adenocarcinoma of the pancreatic head is planned for induction chemotherapy with FOLFIRINOX, with scheduled restaging after seven cycles (14 weeks; 98 days). Radiation therapy is expected to take place 1 week (7 days) after completion of NAC, while surgery follows two weeks (14 days) after all neoadjuvant therapies. Her score, prior to receipt of any NAC is thus, 5.2 points, corresponding to a baseline probability of ypN0 of approximately 11.0%. Following receipt of her initial NAC, restaging imaging at week 14 shows stable primary disease, with a new score of 7.4 and probability of 20.0%. Here, the nomogram informs the decision to proceed with at least two additional cycles of NAC (4 weeks; 28 days) as those who receive 13 – 16 weeks of NAC with cT2 disease are more prone to ypN+. Further, those receiving less than 12 weeks of NAC may be more prone to ypN+ if ENI is subsequently employed, and use of 4 cycles (8 weeks, 56 days) could potentiate the effects of ENI given a total NAC duration ≥ 21 weeks. An additional four cycles are successfully delivered with new imaging showing stable disease, yielding an updated nomogram score of 10.8 points with probability now 36.0% for ypN0. Here, a treating radiation oncologist may then determine the effect of various pancreas and ENI dose regimens given the current neoadjuvant treatment. For example, an elective dose of 25 Gy in 5 fractions (BED10 = 37.5 Gy) across 5 calendar days shows the patient would have a score of 13.2 points (Probability = 36.0%), whereas 45 Gy in 25 fractions (BED10 = 53.1 Gy) over 33 calendar days would raise her score to 14.0 points (Probability = 56.0%). Her treatment takes a total of 40 calendar days given use of a primary boost, bringing her final score to 14.0 points (Probability = 56.0%). With a threshold probability of 52.8%, this score would place her in the cohort predicted to have lower risk of ypN+ disease after ENI.
